# Anti-allergic drug azelastine suppresses colon tumorigenesis by directly targeting ARF1 to inhibit IQGAP1-ERK-Drp1-mediated mitochondrial fission

**DOI:** 10.7150/thno.48698

**Published:** 2021-01-01

**Authors:** Hui-Fang Hu, Wen Wen Xu, Yang-Jia Li, Yan He, Wei-Xia Zhang, Long Liao, Qi-Hua Zhang, Lei Han, Xing-Feng Yin, Xiao-Xu Zhao, Yun-Long Pan, Bin Li, Qing-Yu He

**Affiliations:** 1 MOE Key Laboratory of Tumor Molecular Biology and Key Laboratory of Functional Protein Research of Guangdong Higher Education Institutes, Institute of Life and Health Engineering, Jinan University, Guangzhou, China.; 2 MOE Key Laboratory of Tumor Molecular Biology and Guangdong Provincial Key Laboratory of Bioengineering Medicine, National Engineering Research Center of Genetic Medicine, Institute of Biomedicine, College of Life Science and Technology, Jinan University, Guangzhou, China.; 3 MOE Key Laboratory of Tumor Molecular Biology and Department of General Surgery, First-Affiliated Hospital, Jinan University, Guangzhou, China.

**Keywords:** colorectal cancer, azelastine, drug repurposing, ARF1, mitochondrial fission

## Abstract

This study aimed to screen novel anticancer strategies from FDA-approved non-cancer drugs and identify potential biomarkers and therapeutic targets for colorectal cancer (CRC).

**Methods:** A library consisting of 1056 FDA-approved drugs was screened for anticancer agents. WST-1, colony-formation, flow cytometry, and tumor xenograft assays were used to determine the anticancer effect of azelastine. Quantitative proteomics, confocal imaging, Western blotting and JC-1 assays were performed to examine the effects on mitochondrial pathways. The target protein of azelastine was analyzed and confirmed by DARTS, WST-1, Biacore and tumor xenograft assays. Immunohistochemistry, gain- and loss-of-function experiments, WST-1, colony-formation, immunoprecipitation, and tumor xenograft assays were used to examine the functional and clinical significance of ARF1 in colon tumorigenesis.

**Results:** Azelastine, a current anti-allergic drug, was found to exert a significant inhibitory effect on CRC cell proliferation *in vitro* and *in vivo*, but not on ARF1-deficient or ARF1-T48S mutant cells. ARF1 was identified as a direct target of azelastine. High ARF1 expression was associated with advanced stages and poor survival of CRC. ARF1 promoted colon tumorigenesis through its interaction with IQGAP1 and subsequent activation of ERK signaling and mitochondrial fission by enhancing the interaction of IQGAP1 with MEK and ERK. Mechanistically, azelastine bound to Thr-48 in ARF1 and repressed its activity, decreasing Drp1 phosphorylation. This, in turn, inhibited mitochondrial fission and suppressed colon tumorigenesis by blocking IQGAP1-ERK signaling.

**Conclusions:** This study provides the first evidence that azelastine may be novel therapeutics for CRC treatment. ARF1 promotes colon tumorigenesis, representing a promising biomarker and therapeutic target in CRC.

## Introduction

Colorectal cancer (CRC) is one of the most common malignant tumors and ranks fifth in mortality rate worldwide 1. Despite remarkable advances in surgery, radiation, and chemotherapy, CRC still carries a poor prognosis due to late diagnosis, chemoresistance, and metastasis. Chemotherapeutic agents have serious adverse effects because of the damage to normal tissues, and the tumors always develop resistance to the drugs after a period of treatment. Therefore, there is an urgent need to identify novel cancer targets and develop useful anticancer agents with favorable therapeutic efficacy and low toxicity.

The discovery of new anticancer drugs is a systematic process with high investment and high risk. The drugs currently used in the clinic for various diseases are gaining increasing attention because of their detailed physicochemical characterization and pharmacokinetics as well as evidence of drug safety. For example, warfarin, an anti-coagulation medication, has been used for treatment and risk reduction in prostate, lung, and breast cancers 2. Similarly, leflunomide, a rheumatoid arthritis medication, has been used to treat melanoma in mice 3. Thus, it may be a faster and more effective strategy to repurpose existing drugs, particularly the USA Food and Drug Administration (FDA)-approved drugs for cancer treatment. In this study, by employing a small molecule library consisting of 1056 FDA-approved drugs in high-throughput screening, we identified azelastine, an anti-allergic drug used for relieving nasal symptoms, as a novel anticancer agent. Azelastine prevents asthma by blocking histamine binding to the H1 receptor, inhibiting histamine and leukotriene release from mast cells, and blocks phosphodiesterase activity 4-6. However, the anticancer effect of azelastine has not been reported so far, and its pharmacological mechanism is unknown.

The identification of direct drug targets is important for understanding the underlying mechanisms as well as clinical implications. The Drug Affinity Responsive Target Stability (DARTS) assay relies on the concept that proteolytic enzymes may not degrade the target proteins interacting with drugs and thus can be identified by mass spectrometry 7. In this context, data-independent acquisition (DIA) quantitative proteomics, a new type of mass spectrometry data acquisition method offering high throughput quantitative precision and high repeatability 8-10, has been applied to identify protein-metabolite interactions 11 and search for small molecule target proteins when coupled with the DARTS assay 12, 13. In this study, we utilized DIA proteomics to illustrate that azelastine exerts its anticancer effects by affecting the ERK pathway and mitochondrial function. By combining DIA proteomics with the DARTS assay, we identified ADP-ribosylation factor 1 (ARF1) as a direct target of azelastine.

Mitochondrial dysfunction has significant consequences for apoptosis, metabolism, and cancer development. Emerging evidence suggests that the dynamic balance between mitochondrial fission and fusion is essential for maintaining the morphology, distribution, and function of mitochondria 14, 15. Dynamin-related protein 1 (Drp1), a dynamin-like protein required for mitochondrial fission, has been reported to be regulated by the ERK pathway 16. However, the underlying mechanisms in Drp1 regulation and mitochondrial fission remain to be elucidated. Interestingly, ARF1 is highly expressed in a variety of malignant tumors 17, 18 and has been reported to activate the ERK pathway in breast cancer 19. However, the biological function of ARF1 in CRC is still unknown. To understand how azelastine targets ARF1 and exerts its antitumor effects, we investigated ARF1's role in colon tumorigenesis and found that ARF1 activates the IQ-domain GTPase-activating protein 1 (IQGAP1)-mediated ERK pathway to regulate Drp1 phosphorylation. A series of functional assays were performed to illustrate that azelastine inhibited mitochondrial fission to exert anticancer activity by blocking ARF1-IQGAP1-ERK-Drp1 signaling* in vitro* and *in vivo*.

## Materials and Methods

### Cell culture and drug

Human CRC cell lines were purchased from ATCC (Rockville, MD), DLD1and HCT116 cells were cultured in RPMI 1640, and HT20 and RKO cells were cultured in DMEM (Thermo Fisher Scientific, San Jose, CA) at 37°C in 5% CO_2_. Azelastine was acquired from Selleck Chemicals (Huston, TX) and dissolved in H_2_O.

### Tissue Microarray and Immunohistochemistry

Immunohistochemistry was performed as previously described 20. The tissue microarrays containing 202 cases of CRC tissues and 158 matched adjacent normal tissues (Shanghai Outdo Biotech, Shanghai, China) were used to analyze ARF1 expression and its correlation with clinicopathological parameters. In brief, the slides were blocked with normal serum and then incubated with anti-Ki-67 (Dako, Mississauga, ON, Canada), anti-p-ERK, or anti-ARF1 antibodies overnight at 4°C, followed by matching biotinylated secondary antibodies and peroxidase-conjugated avidin-biotin complex (Dako). 3,3′-diaminobenzidine (Dako) was used as a chromogen to visualize the immunostaining, and the sections were counterstained with hematoxylin. A scale of 1 (negative) to 2 (weak) representing low expression and a scale of 3 (moderate) to 4 (strong) representing high expression was used to grade the intensity of ARF1 and p-ERK staining.

### Plasmids, transfection, infection, and CRISPR/Cas9-mediated gene knockout

Full-length ARF1 was amplified and cloned into the prokaryotic expression plasmid pET-28b (Novagen, Madison, WI). The siRNA against ARF1, the transient and stable ARF1-overexpressing plasmids, as well as plasmids expressing the shRNA and sgRNA against ARF1, were obtained from TransheepBio (Shanghai, China). The inducible ARF1-knockdown plasmid, IQGAP1-overexpressing plasmid, and the plasmid expressing sgRNA against IQGAP1 were purchased from IGEbio (Guangzhou, China). Using the ClonExpress II One Step Cloning Kit (Vazyme, Nanjing, China), the plasmids pcDNA3.1-IQGAP1-∆RGCT-flag, pcDNA3.1-IQGAP1-∆RGCT/GRD-flag, and pcDNA3.1-IQGAP1-∆RGCT/GRD/IQ-flag were generated by PCR amplification from pcDNA3.1-IQGAP1 and cloned into the pcDNA3.1 vector. The mutant constructs for pcDNA3.1-ARF1^T48S^, pcDNA3.1-ARF1^C159G^, and pET28b-ARF1^T48S^ were created by the* Fast* Mutagenesis System (TransGen Biotech, Beijing, China). Transfection and establishment of stable cell lines were carried out as described previously 21. The sequences of siRNA and primers for cloning and mutation are listed in Table S1.

### Cell viability assay

The CRC cell viability was measured by WST-1 Cell Proliferation and Cytotoxicity Assay Kit (Beyotime Biotechnology, Shanghai, China), as previously described 22. WST-1 was added, and cells were incubated for 2 h at 37°C, and the absorbance at 450 nm was measured on an automated microplate spectrophotometer (BioTek Instruments, Winooski, VT).

### Colony formation assay

CRC cells were plated in 6-well or 12-well plates and treated with different azelastine concentrations for 14 days, as previously described 20. Methanol and 1% crystal violet were used to fix and stain the cells, and the colonies formed was quantified.

### Soft agar Colony Formation Assay

The culture medium was mixed with 1.2% agar at a ratio of 1:1, added to a 12-well plate, and solidified at room temperature. The cells were resuspended in the culture medium containing 0.3% agar and added on top of the base agar layer. Colonies were photographed 2 weeks later and counted for analysis of anchorage-independent growth ability.

### Annexin V-FITC/PI staining assay

Cell apoptosis was detected by the Annexin V-FITC/PI Apoptosis Detection kit (KeyGen, Jiangsu, China) 23. Cells were suspended in binding buffer, stained with annexin V-FITC and propidium iodide (PI) for 15 min, and analyzed on a BD Accuri C6 flow cytometer (BD Biosciences, San Diego, CA).

### TdT-mediated dUTP nick-end labeling assay

The TdT-mediated dUTP nick-end labeling (TUNEL) *in situ* cell death detection kit Fluorescein (Roche Diagnostics, Mannheim, Germany) was used to determine cell apoptosis. In brief, the slides were deparaffinized and rehydrated, then incubated with TUNEL reaction mixture for 1 h, followed by DAPI staining. The images were taken, and the percentage of apoptotic cells was counted.

### Transmission electron microscope (TEM)

The cells were fixed with TEM fixative (Wuhan Servicebio Technology, Wuhan, China) at 4°C for 4 h, then pre-embedded in 1% agarose, and fixed with 1% osmium tetroxide. After dehydration at room temperature using ethanol, the cells were embedded in Poly/Bed 812 resin, followed by polymerization at 65°C. The ultrathin section was stained with 2% uranium acetate saturated alcohol solution and 2.6% lead citrate. TEM (HT7700, HITACHI, Fukuoka, Japan) was used to take images for morphological analysis.

### Measurement of the mitochondrial membrane potential

A JC-1 assay kit (Beyotime Biotechnology) was used to measure mitochondrial membrane potential, as previously described 24. Cells were treated with indicated concentrations of azelastine for 48 h, stained with JC-1 at 37°C for 20 min, and analyzed by flow cytometry. The change in mitochondrial polarization was calculated as the red /green fluorescence intensity ratio.

### Quantification of mitochondrial morphology

MitoTracker® Mitochondrion-Selective Probe (Invitrogen, Gaithersburg, MD) was used to detect the morphology of mitochondria. Cells were incubated with the staining solution containing MitoTracker® probe (100 nM) at 37°C for 30 min and fixed with 4% formaldehyde, followed by DAPI staining. The images of each well were obtained in three different fields by confocal microscopy. The morphology of mitochondria was analyzed using Image J with mitochondrial network analysis toolset (MiNA).

### Mass spectrometry and bioinformatics analyses

Protein digestion and mass spectrometry analysis were executed as previously described 24. In brief, HT29 cells treated with azelastine for 48 h were lysed with a lysis buffer. Proteins were digested with trypsin, vacuum-freeze-dried, and resuspended in anhydrous acetonitrile solution, then desalted with MonoTIPTM C18 Pipette Tip (GL Sciences, Tokyo, Japan). Peptide samples were analyzed with an Orbitrap Fusion Lumos mass spectrometer (Thermo Fisher Scientific). Then raw data were analyzed using Proteome Discoverer (Thermo Fisher Scientific) and Spectronaut (Omicsolution Co., Ltd., Shanghai, China) software. Protein and peptide FDRs were set to 1%, and Ingenuity Pathway Analysis (IPA, Ingenuity Systems, Redwood City, CA) was used to analyze the differentially expressed proteins.

### Western blotting

Cell lysates were prepared as previously described 25, an equal volume of the loading buffer was added and boiled. Protein samples were loaded onto a sodium dodecyl sulfate (SDS)-PAGE gel, electrophoresed, and subsequently transferred to a PVDF membrane (Millipore, Bedford, MA). The membrane was incubated with the primary antibody for 2 h, and then incubated with the corresponding secondary antibody for 1 h. After washing with PBS, signals were detected using the ECL substrate (Bio-Rad, Hercules, CA). The primary antibodies used included caspase-3, cleaved caspase-3, Bcl-2, Bax, ERK, p-ERK (Cell Signaling Technology), Bcl-xL (Abcam, Cambridge, MA), ARF1, IQGAP1, p-Drp1 (Proteintech, Chicago, IL) and actin (Santa Cruz Biotechnology, Santa Cruz, CA).

### Co-immunoprecipitation (Co-IP)

Immunoprecipitation was performed as described previously 26. The cell lysates were incubated with IgG (Santa Cruz Biotechnology) and protein A/G Sepharose beads (Invitrogen) at 4ºC for 1 h. The supernatant was mixed with appropriate primary antibody overnight at 4ºC, followed by a 4 h incubation with protein A/G Sepharose beads. After washing with PBS and lysis buffer, the beads were mixed with 5 × SDS/PAGE loading buffer for Western blotting analysis.

### Drug Affinity Responsive Target Stability (DARTS) assay

Cell lysates were mixed with azelastine for 30 min and incubated with proteinase K for 5 min at 25°C according to the mass ratio of 1:100. The reaction was stopped with a final concentration of 5% sodium deoxycholate (Sigma Aldrich, ST, Louis) for 3 min at 98°C, and proteins were identified by mass spectrometry and bioinformatics analysis.

### ARF1 activation assay

The ARF1 activity in CRC cells and tissues was measured by an ARF1 activation assay kit (Abcam) following the manufacturer's instruction. GGA3 PBD Agarose beads were used to pull-down the active form of ARF1 from cell and tissue lysates, and GTP-ARF1 in the immunoprecipitates was analyzed by Western blotting using an ARF1 antibody.

### Purification of ARF1 protein

The pET-28b vector was used to construct the plasmids pET-28b-ARF1 and pET-28b-ARF1^T48S^, expressing wild-type and mutant histidine (His)-tagged ARF1 fusion proteins, according to a previously described procedure 26. The plasmids were transformed into E coli BL21 (DE3) star cells, and 0.5 mM isopropyl β-D-thiogalactopyranoside (IPTG) was added for 12 h to induce the expression of ARF1-His protein when the optical density at 600 nm of bacterial culture reached about 0.6. The bacteria were lysed by sonication and His-tagged ARF1 fusion protein was isolated by using a His-binding column (Beyotime Biotechnology) and analyzed by Western blotting.

### Biacore assay

The interaction between azelastine and ARF1 was analyzed with a Biacore X100 system (GE Healthcare Life Sciences). In brief, the purified protein solution was adjusted to an appropriate pH value with acetic acid. ARF1 was coupled with CM7 chip (GE Healthcare Life Sciences), which was pre-balanced with PBS containing 0.4% P20 according to the manufacturer's instructions. Different concentrations of azelastine (150, 75, 37.5, 18.75, and 9.375 μM) were diluted with running buffer, and the samples were loaded to detect the response values. Biacore analysis software was used to fit the curve, and the Kd value of the small molecule and the protein was obtained.

### Tumor xenograft experiments

Details of tumor xenograft experiments were previously described 27. BALB/c nude mice aged 6-8 weeks were cared for under standard conditions according to institutional guidelines. All animal experiments were approved by the Ethics Committee for Animal Experiments of Jinan University. In brief, cells were digested and resuspended in PBS, then mixed with the equal volumes of Matrigel, and subcutaneously injected into the flanks of nude mice. Tumor size and body-weight of mice were measured every three days, and the tumor volume was calculated as V = (length × width^2^)/2. In the treatment experiments, when the tumors reached 5 mm in diameter, the mice were randomly divided into treatment and control groups. The treatment groups received oral gavage of azelastine (10 mg/kg or 20 mg/kg) every two days, whereas the control group received vehicle. At the end of the experiment, the tumors, lungs, livers, and kidneys were collected for histological, immunohistochemical, and Western blotting analyses. Alanine aminotransferase (ALT) and aspartate aminotransferase (AST) in mice serum were measured using commercial kits (HuiLi Biotech Ltd., Changchun, China).

### Statistical analysis

*In vitro* experiments were performed three times. The data were expressed as the mean ± SD and analyzed by *t*-test. The association between ARF1 expression and patients' survival was analyzed by the Kaplan-Meier method and the log-rank test was used to compare the statistical difference. *P* < 0.05 was considered statistically significant.

## Results

### Anti-allergic drug azelastine is a potential anticancer agent

**Figure 1:**
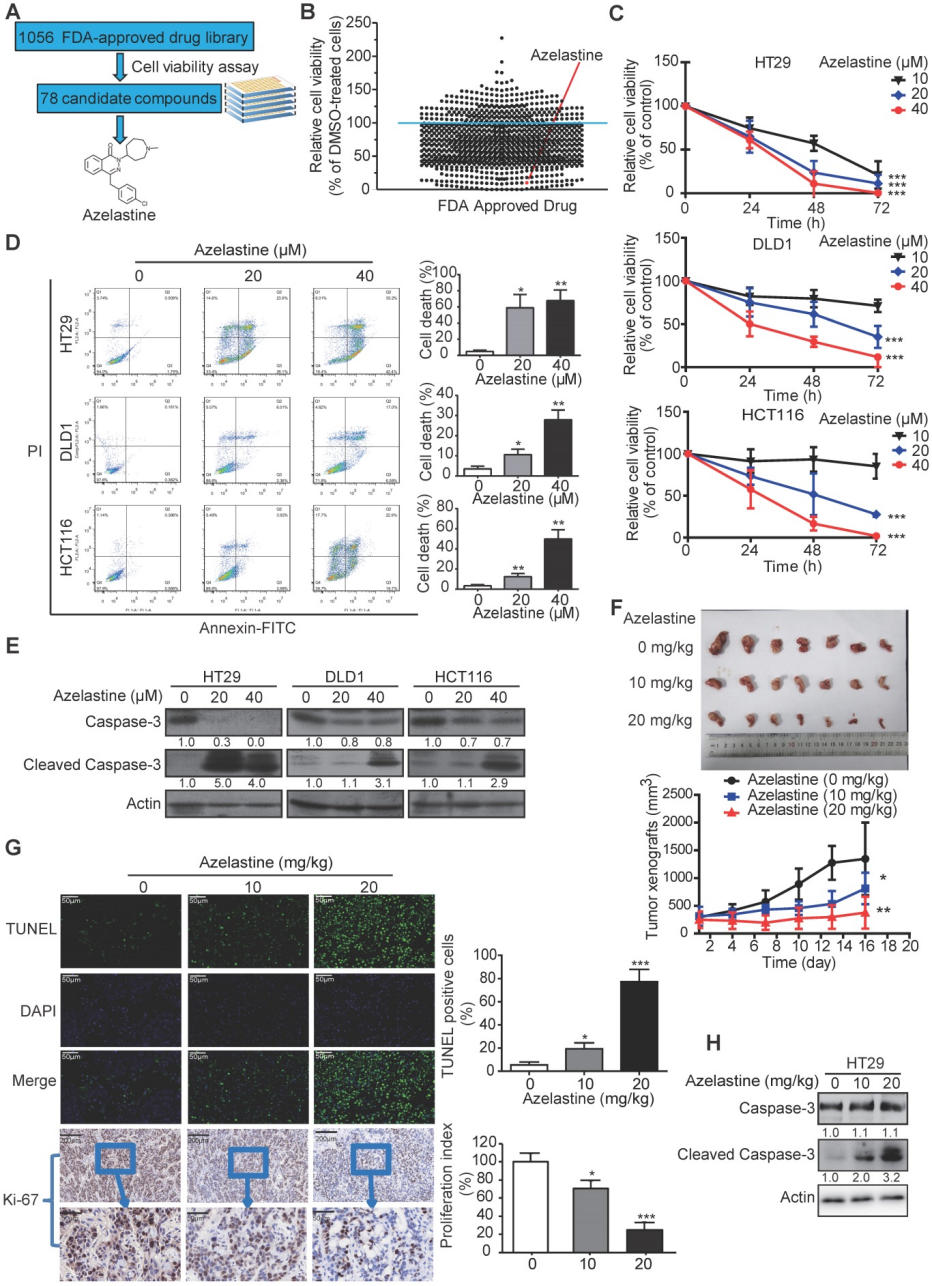
** Azelastine inhibits CRC cell proliferation *in vitro* and *in vivo.* (A)** the experimental schema for identifying anticancer drugs with an FDA-approved drug library. **(B)** Cell viability of HT29 cells, treated with 1056 drugs (10 µM) individually for 72 h, was measured by WST-1. The red point represents azelastine. **(C)** Comparison of the cell viability of HT29, DLD1, and HCT116 cells treated with different concentrations of azelastine for up to 72 h. **(D-E)** HT29, DLD1, and HCT116 cells were treated with various azelastine concentrations for 48 h, and apoptotic cells were analyzed by Annexin V-FITC/PI double-staining **(D)**, and caspase-3 and cleaved caspase-3 expression levels were compared **(E)**. **(F)** Mice bearing HT29-derived tumor xenografts were orally administrated with azelastine (10 mg/kg or 20 mg/kg) or vehicle every two days. Images showed that azelastine exerted an inhibitory effect on the growth of HT29-derived tumor xenografts (n = 7).** (G)** Tumor cell apoptosis analyzed by TUNEL assay and quantification of Ki-67 proliferation index in tumors. **(H)** Comparison of Caspase-3 and Cleaved Caspase-3 expression in the azelastine-treated tumor xenografts. Bars, SD; *, *P* < 0.05; **, *P* < 0.01; ***, *P* < 0.001.

To screen the FDA-approved drugs with new indications in oncology, we took advantage of a library comprising of 1056 FDA-approved drugs (Table S4), and used CRC as a cancer model. CRC cells were treated with the 1056 small molecules individually or DMSO control for 72 h, followed by the cell viability assay to determine each compound's inhibitory effect (Figure 1A). As shown in Figure 1B, a total of 78 drugs (Table S2) exhibited an inhibitory effect (> 80%) on cancer cell growth. As a positive control, motolimod (VTX-2337), the commonly used drug for treating head and neck squamous cell carcinoma in the clinic 28, exhibited anti-tumor effect as expected, indicating the validity of our strategy for identifying novel compounds with anticancer ability. Among the 78 candidates, we focused on azelastine, an anti-allergic drug, which has not been linked to cancer treatment.

### Azelastine inhibits proliferation of CRC cells *in vitro* and* in vivo*

HT29, DLD1, and HCT116 cells were exposed to different azelastine concentrations for up to 72 h followed by the WST-1 assay. As displayed in Figure 1C, CRC cell viability was significantly reduced with increasing concentration of azelastine. Besides, azelastine significantly decreased both anchorage-dependent and -independent colony formation abilities of CRC cells, as determined by the colony formation and soft agar assays (Figure S1A-B). The annexin V-FITC/PI double-staining assay was used to analyze azelastine's effect on apoptosis. The results indicated apoptosis induction in a dose-dependent manner in HT29, DLD1, and HCT116 cells (Figure 1D), which was also confirmed by the increased cleaved caspase-3 expression upon azelastine treatment (Figure 1E).

To examine the therapeutic potential of azelastine in cancer treatment, nude mice were subcutaneously injected with HT29 cells and orally administrated with azelastine. As shown in Figure 1F, azelastine significantly inhibited tumor burden by 40.7% and 72.1% in 10 mg/kg and 20 mg/kg azelastine-treated mice. No noticeable difference in the bodyweight of mice was observed between the treatment and control groups (Figure S1C). The TUNEL assay and Ki-67 immunohistochemical analysis indicated that azelastine significantly induced apoptosis and inhibited tumor cell proliferation (Figure 1G). Histological examination of the liver, kidney, and lung revealed no toxic effects of azelastine treatment (Figure S1D). Consistent with the *in vitro* data, increased cleaved caspase-3 expression was observed in tumor xenografts treated with azelastine (Figure 1H). These data revealed that azelastine has a potent antitumor effect in CRC *in vitro* and* in vivo.*

### Proteomics analysis indicates mitochondrial dysfunction and involvement of ERK signaling in the anticancer mechanism of azelastine

**Figure 2:**
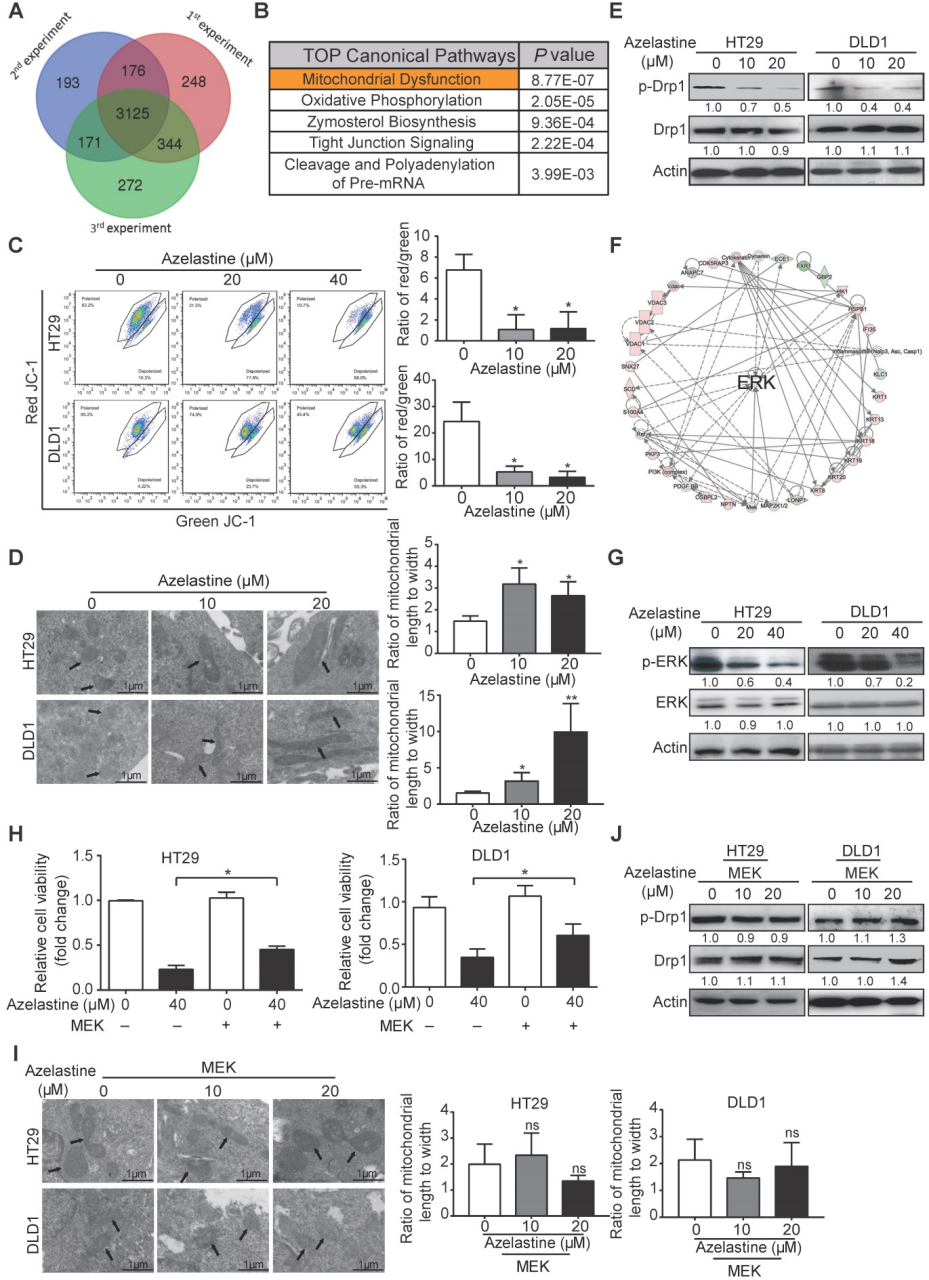
** Proteomics suggests the involvement of mitochondrial dysfunction and ERK signaling in the antitumor mechanism of azelastine. (A)** Overlapping proteins identified in triplicate. **(B)** Azelastine-regulated proteins analyzed by IPA. **(C)** Integrity of mitochondrial membrane determined by JC-1 assay in HT29 and DLD1 cells treated with azelastine for 48 h. **(D-E)** CRC cells were treated with indicated concentrations of azelastine for 48 h, and TEM was performed to compare the morphology of mitochondria (D), and p-Drp1 expression detected by Western blotting (E). **(F)** Network analysis suggested the regulation of ERK signaling by azelastine. **(G)** ERK and p-ERK expression was detected in HT29 and DLD1 cells treated with indicated concentrations of azelastine for 48 h. **(H-J)** CRC cells overexpressing MEK were treated with indicated concentrations of azelastine for 48 h; WST-1 and TEM were performed to compare the cell viability (H), morphology of mitochondria (I), and p-Drp1 expression (J). Bars, SD; *, *P* < 0.05; **, *P* < 0.01; ***, *P* < 0.001.

To explore its mechanism of action, HT29 cells were treated with azelastine or H_2_O for 48 h followed by DIA-mass spectrometry. As shown in the Venn diagram, 3125 proteins with quantitative information were identified in triplicate experiments (Figure 2A). The power law global error model (PLGEM) was used to determine the protein abundance, with a slope of 1 and an adjusted r^2^ of 0.989 (Pearson r = 0.82) (Figure S2A). As presented in Figure S2B, the data had a fitted normal distribution of the residual standard deviations between modeled and actual values. The residuals were distributed evenly and were independent of the rank of mean abundances (Figure S2C-D).

A total of 164 differentially expressed proteins were identified in triplicate experiments, including 56 down-regulated and 108 up-regulated proteins (FC ≥ 1.5, *p-value* ≤ 0.05, Table S3). Subsequently, the differentially expressed proteins were uploaded to the Ingenuity Pathway Analysis (IPA) software to analyze signaling pathways affected by azelastine. Among the predicted top five canonical pathways, mitochondrial dysfunction ranked first with a *P*-value of 8.77 × 10^-7^ (Figure 2B). We determined the integrity of the mitochondrial membrane by the JC-1 assay, in which red and green fluorescence represent healthy and damaged mitochondria, respectively. Figure 2C displays a dose-dependent decrease of red/green fluorescence ratio in azelastine-treated HT29 and DLD1 cells, suggesting that azelastine induces mitochondrial dysfunction and apoptosis.

The mitochondrial dysfunction was further confirmed by decreased expression levels of Bcl-xL and Bcl-2 proteins involved in the mitochondrial pathway of azelastine-treated CRC cells (Figure S3). Furthermore, the morphology of mitochondria, detected by TEM and immunofluorescence staining, exhibited weaker mitochondrion fission ability in azelastine-treated CRC cells (Figure 2D and Figure S4A). Western blotting data also showed that azelastine significantly decreased the expression of p-Drp1, the mitochondrial fission marker, in HT29 and DLD1 cells (Figure 2E), suggesting that azelastine inhibits mitochondrial fission to induce apoptosis in CRC cells.

Network analysis of azelastine-regulated proteins indicated that ERK signaling might play a central role in the anticancer effect of azelastine (Figure 2F). Western blotting verified that p-ERK expression was significantly decreased in azelastine-treated CRC cells (Figure 2G and Figures S4B). To investigate whether ERK signaling mediated the bioactivity of azelastine, HT29, DLD1, and HCT116 cells were transfected with MEK-overexpressing plasmid and vector control and cell viability was compared upon azelastine treatment. WST-1 assay showed that activation of the ERK pathway significantly abrogated the inhibitory effect of azelastine on CRC cell proliferation (Figures S4C-D and Figure 2H). We also observed that ERK re-activation not only rescued azelastine's effect on mitochondrial fission (Figure 2I and Figures S4E), but also abolished the azelastine-induced p-Drp1 down-regulation (Figure 2J and Figures S4F). We compared the anticancer effect of rotenone, a mitochondrial complex I inhibitor, with azelastine, and the results showed that azelastine's effect on CRC cell proliferation was similar to that of rotenone (Figure S4G). Taken together, azelastine might inhibit mitochondrial fission via the ERK signaling pathway to suppress colon tumorigenesis.

### ARF1 is a direct target of azelastine

We next examined whether azelastine exerted anticancer effects through the histamine receptor H1 (HRH1), the known target protein of azelastine in its original anti-allergic indication 29. HRH1-deficient CRC cells were successfully established by using CRISPR/Cas9 technology (Figure S5A) and compared with control cells for their sensitivity to azelastine. As shown in Figure S5B, knockout of HRH1 could not eliminate the suppressive effect of azelastine in HT29 and HCT116 cells, indicating that HRH1 is not the target protein of azelastine in its anticancer function.

Based on the concept that a protein is resistant to proteolysis when it binds to a ligand such as a small molecule compound, DARTS technology was used to identify azelastine's actual target protein related to its oncological property (Figure S5C). Mass spectrometric analysis of the peptide fragments from the DARTS experiment identified ARF1 as a candidate target protein of azelastine. We then established ARF1-deficient HT29 and DLD1 cell lines for validation and found that ARF1 knockout significantly attenuated the inhibitory effect of azelastine on CRC cell proliferation (Figure S5D-E), illustrating that azelastine directly targeted ARF1 to suppress CRC growth.

Moreover, the proliferation of CRC cells treated with mitochondrial division inhibitor-1 (Mdivi-1) was analyzed. The results showed that the inhibitory effect of azelastine on CRC cell proliferation was significantly abrogated by Mdivi-1 (Figure S5F), suggesting that azelastine mainly exerts its bioactivity through inhibition of ARF1-mediated mitochondrial fission.

### ARF1 promotes colon tumorigenesis by inducing ERK-mediated mitochondrial fission *in vitro* and* in vivo*

**Figure 3:**
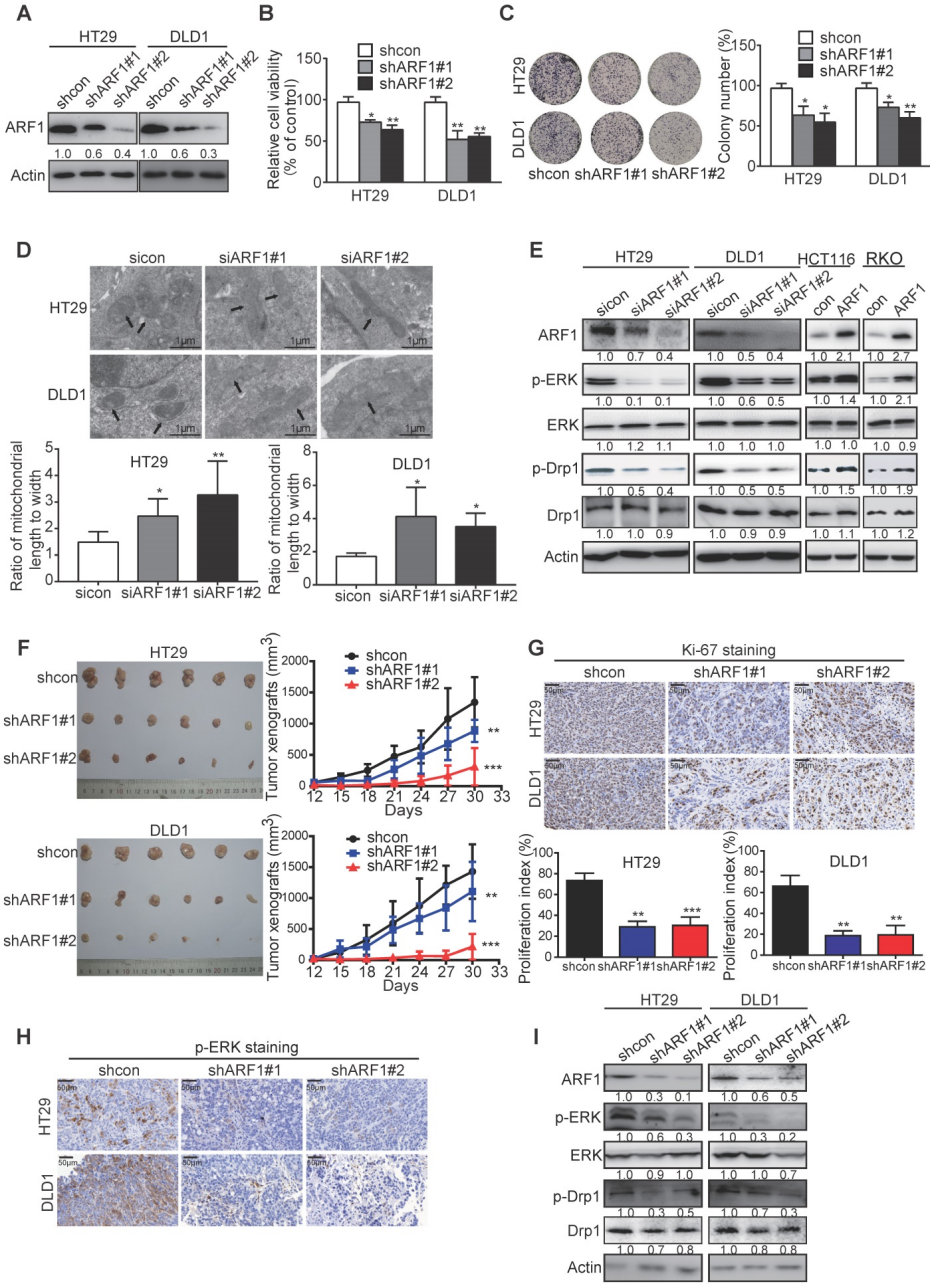
** ARF1 promotes colon tumorigenesis by inducing ERK-mediated mitochondrial fission *in vitro* and* in vivo.* (A)** Successful knockdown of ARF1 in HT29 and DLD1 cells. **(B-C)** Effect of ARF1 knockdown on HT29 and DLD1 cell proliferation (B) and colony formation (C). **(D)** Comparison of the morphology of mitochondria in ARF1-knockdown HT29 and DLD1 cells and control cells. **(E)** Effect of manipulating ARF1 expression on p-ERK and p-Drp1 expression levels. **(F)** Images of tumors and tumor curves showing significant growth inhibition of HT29- and DLD1-derived tumor xenografts (n = 6) by ARF1 knockdown. **(G)** Quantification of Ki-67 proliferation index in tumors. **(H)** Immunohistochemical analysis of p-ERK expression in tumors. **(I)** p-Drp1 and p-ERK expression in tumors. Bars, SD; *, *P* < 0.05; **, *P* < 0.01; ***, *P* < 0.001.

The biological function of ARF1 in CRC has not been reported previously. Stable ARF1-overexpressing cell lines were established using HCT116 and RKO cells, designated as HCT116-ARF1 and RKO-ARF1 (Figure S6A). WST-1 assay showed that the ectopic expression of ARF1 markedly promoted CRC cell proliferation (Figure S6B). Also, as shown in Figure S6C-D, anchorage-dependent and -independent growth abilities significantly increased in the ARF1-overexpressing stable cell lines. To explore whether ARF1 promoted cell proliferation via ERK signaling in CRC, the expression of p-ERK was examined by Western blotting and found to be increased in RKO-ARF1 and HCT116-ARF1 cells (Figure S6E). MEK inhibitor U0126 markedly abolished ARF1 overexpression effects on p-ERK expression, proliferation, and 2D and 3D colony formation in CRC cells (Figure S6F-H). In loss-of-function experiments, stable and inducible ARF1-knockdown HT29 and DLD1 cell lines were established. ARF1 knockdown with two different shRNA sequences significantly reduced HT29 and DLD1 cell proliferation and colony formation (Figure 3A-C and Figure S6I-K). More importantly, ARF1 knockdown repressed the mitochondrial fission and decreased the p-ERK and p-Drp1 expression levels in CRC cells (Figure 3D-E and Figure S6L).

ARF1-knockdown stable cell lines, HT29-shARF#1 and HT29-shARF#2 as well as DLD1-shARF1#1 and DLD1-shARF1#2, were subcutaneously injected into flanks of nude mice to establish tumor xenografts, and the tumor volumes were monitored. The ARF1-knockdown CRC cells formed smaller tumors than control cells, with decreases of 35.7% and 75.6% for HT29-derived tumors and 31.8% and 85.3% for DLD1-derived tumors (Figure 3F). Ki-67 and p-ERK staining data also confirmed that cell proliferation and ERK signaling were significantly inhibited in ARF1-knockdown cells (Figure 3G-H). Consistent with the *in vitro* data, Western blotting analysis of tumor xenografts showed ARF1 knockdown inactivated ERK signaling and decreased p-Drp1 expression (Figure 3I). Collectively, *in vitro* and* in vivo* assays demonstrated that ARF1 induces mitochondrial fission via the ERK signaling pathway to promote colon tumorigenesis.

### ARF1 expression is of clinical significance in CRC

**Figure 4:**
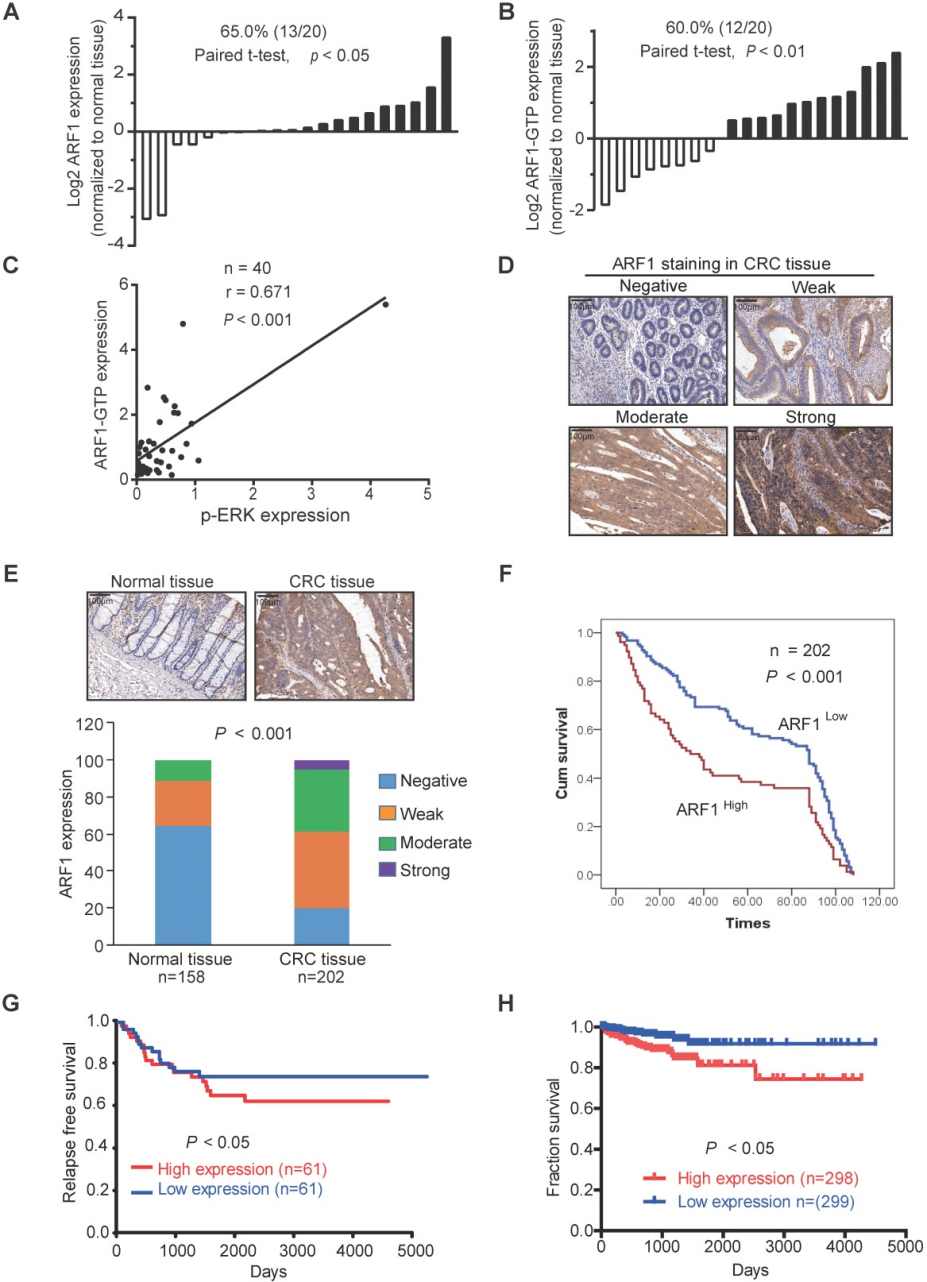
** Clinical significance of ARF1 in CRC. (A-B)** Graph showing that the expression of ARF1 and ARF1-GTP was higher in tumor tissues than the corresponding adjacent normal tissues. **(C)** Positive correlation between ARF1-GTP and p-ERK expressions in CRC tissues. **(D)** Different scores of ARF1 staining in CRC. **(E)** Comparison of ARF1 expression in primary tumors and matched normal tissues by immunohistochemical staining. **(F)** Overall survival of 202 CRC patients analyzed by Kaplan-Meier analysis stratified according to tumor ARF1 expression. **(G-H)** Association between ARF1 expression and CRC patients' survival determined in the GEO database **(G)** and starBase v3.0 **(H)**. Bars, SD; *, *P* < 0.05; **, *P* < 0.01; ***, *P* < 0.001.

To determine the clinical significance of ARF1, we analyzed ARF1 expression in 20 pairs of CRC tumor tissues and adjacent normal tissues by Western blotting. The majority of tumor cases had a stronger expression of ARF1 (65%) and ARF1-GTP (60%) than adjacent normal tissues (Figure S7A and Figure 4A-B), with a positive ARF1-GTP correlation with p-ERK expression (Figure 4C). A microarray containing 202 cases of CRC tumor tissues and 158 cases of adjacent normal tissues was used to analyze ARF1 expression by immunohistochemistry and determine its correlation with patients' clinicopathological parameters (Figure 4D). As shown in Figure 4E and Table 1, most of the tumor tissues had higher ARF1 expression than adjacent normal tissues. Kaplan-Meier survival analysis revealed significantly shorter survival (median survival = 33.0 months) in patients with high ARF1 expression than those with low ARF1 expression (median survival = 88.0 months) (Log-rank = 11.47, *P* < 0.001, Figure 4F).

Analysis of data from the Gene Expression Omnibus (GEO) Datasets and starBase v3.0 30 also showed that ARF1 expression was correlated with poor survival of CRC patients (Figure 4G-H). Further, as presented in Figure S7B, ARF1 expression was stronger in other cancers than normal tissues, and negatively correlated with patient survival (Figure S7C). These data suggested that ARF1 is a potential diagnostic and prognostic biomarker in CRC.

### ARF1 interacts with IQGAP1 to activate ERK signaling in colon tumorigenesis

**Figure 5:**
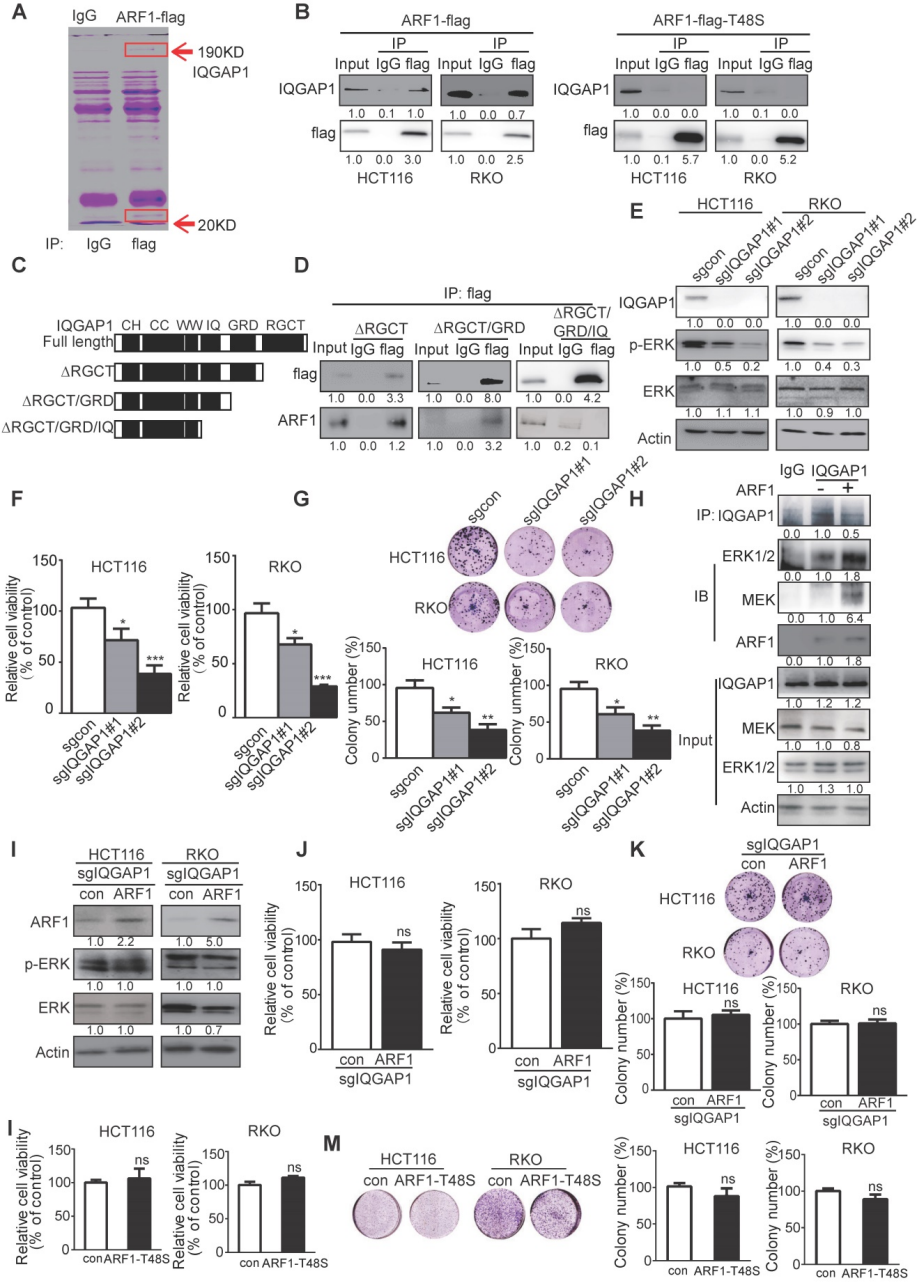
** ARF1 exerts its oncogenic function by interacting with IQGAP1. (A)** ARF1 immunoprecipitates in HCT116 cells were separated on a gel. **(B)** Immunoprecipitation was performed to determine the interaction of IQGAP1 with wild-type ARF1 and ARF1-T48S mutant in HCT116 and RKO cells. **(C)** Schematic showing the truncated mutants of IQGAP1. **(D)** HCT116 cells were transfected with the flag-ARF1 plasmid and the plasmids expressing wild-type IQGAP1 or its truncated mutations. Cell lysates were immunoprecipitated using flag antibody, then bound proteins were analyzed. **(E)** Successful establishment of IQGAP1-deficient HCT116 and RKO cells, and comparison of p-ERK expression by Western blotting. **(F-G)** WST-1 and colony formation assays were used to determine the cell proliferation rate **(F)** and colony-forming abilities **(G)** of IQGAP1-knockout cells and control cells. **(H)** Immunoprecipitation showing that overexpression of ARF1 enhanced the interaction of IQGAP1 with MEK and ERK. **(I-K)** ARF1 was overexpressed in IQGAP1-deficient HCT116 and RKO cells, and p-ERK expression, cell proliferation, and colony-forming ability were compared with control cells by Western blotting **(I),** WST-1 **(J)** and colony formation assays **(K)**, **(L-M)** WST-1 and colony formation assays were performed to determine proliferation and colony formation of HCT116 and RKO cells overexpressing ARF1-T48S mutant. Bars, SD; *, *P* < 0.05; **, *P* < 0.01; ***, *P* < 0.001.

To explore the underlying mechanism by which ARF1 activates ERK signaling, immuno-precipitation combined with mass spectrometry was used to detect ARF1 interacting proteins in CRC cells (Figure 5A). Among the proteins identified, IQGAP1, which has been reported to interact with ARF6 and multiple components of mitogen-activated protein kinase (MAPK1) pathway 31, was of interest. Given that Thr-48 of ARF1 plays a key role in its activation and function 32, we next examined whether this amino acid was essential for IQGAP1 binding. Co-immunoprecipitation indicated that wild-type ARF1 could interact directly with IQGAP1, whereas when we constructed ARF1 with a mutation at Thr-48 to Ser, it decreased the interaction (Figure 5B).

IQGAP1 has several recognizable substrate-binding domains, including Ras-GAP C-terminus (RGCT) (amino acids 1276-1657), Ras-GAP (GRD) (amino acids 1004-1237), isoleucine/glutamine-containing (IQ) (amino acids 745-864), WW (WW) (amino acids 681-710), coiled-coil (CC) (amino acids 160-680), and calponin homology (CH) (amino acids 44-159) domains. We next truncated IQGAP1 to study which domains were required for its interaction with ARF1. As displayed in Figure 5C-D, domain mapping results indicated a diminished interaction between ARF1 and IQGAP1 upon deletion of amino acids 745-1657 (∆IQ/GRD/RGCT), but not amino acids 1004-1657 (∆RGCT/GRD) or 1276-1657 (∆RGCT), suggesting that the IQ domain in IQGAP1 is essential for ARF1 binding.

IQGAP1-knockout was then generated in HCT116 and RKO cells. WST-1 and colony-formation assays demonstrated that IQGAP1 could positively induce ERK signaling and CRC cell proliferation (Figure 5E-G). Since IQGAP1 has been reported to interact with MEK and EKR, we explored whether ARF1 affected this interaction. Immunoprecipitation data showed that ARF1 indeed enhanced IQGAP1 interaction with MEK and ERK (Figure 5H). Next, ARF1 was overexpressed in IQGAP1-deficient cells to examine the significance of IQGAP1 in the functional role of ARF1 in CRC. Western blotting, WST-1, and colony formation assays (Figure 5I-K) indicated that, unlike parental cells, ectopic ARF1 expression could not increase p-ERK expression or cell proliferation in IQGAP1-knockout HCT116 and RKO cells (Figure S6A-C), suggesting that IQGAP1 was essential for the ARF1 function in cancer. Also, overexpression of ARF1-T48S, the ARF1 mutant that lacked binding to IQGAP1, had no effect on HCT116 and RKO cell proliferation, as indicated by WST-1 and colony-formation assays (Figure 5L-M). Collectively, the data showed that ARF1 activates ERK signaling and colon tumorigenesis by interacting with IQGAP1.

### Thr-48 of ARF1 is required for anticancer activity of azelastine

**Figure 6:**
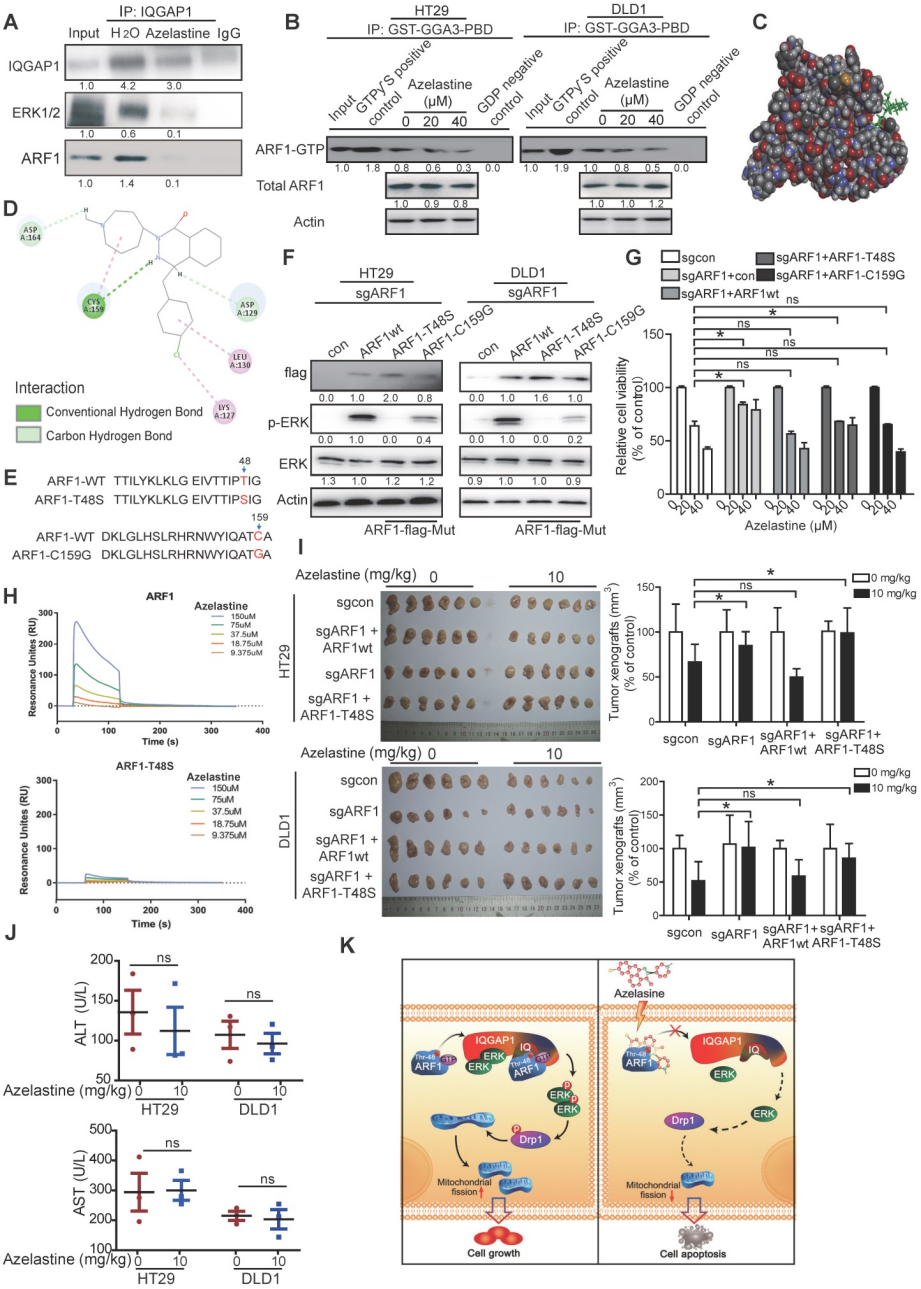
** Thr-48 of ARF1 is required for the anticancer bioactivity of azelastine. (A)** Immunoprecipitation assay showing that the IQGAP1 interactions with ARF1 and ERK were blocked by azelastine.** (B)** ARF1 activation assay was used to measure ARF1 activity in HT29 and DLD1 cells with various concentrations of azelastine treatment for 48 h. **(C)** Molecular docking was used to analyze the potential binding site involved in the combination of azelastine and ARF1. **(D)** 2D diagram showing the bonding of the drug azelastine and the protein ARF1. **(E)** Mutation design of ARF1-T48S and ARF1-C159G. **(F-G)** Wild-type ARF1, ARF1-T48S mutant, or ARF1-C159G mutant was overexpressed in ARF1-deficient HT29 and DLD1 cells, and p-ERK expression was analyzed **(F)**, and the WST-1 assay was performed to compare the sensitivity of CRC cell lines to azelastine treatment** (G)**. **(H)** Biacore results showed that ARF1-T48S mutant protein had significantly weaker interaction with azelastine than wild-type ARF1. **(I)** ARF1-deficient HT29 and DLD1 cells were overexpressing wild-type ARF1 or ARF1-T48S mutantwere used to establish tumor xenografts. The nude mice were orally administrated with azelastine (10 mg/kg) or vehicle every two days. Note that azelastine could not inhibit the ability of ARF1-deficient CRC cells to form tumors, and the antitumor effect of azelastine was recovered when the cells were re-overexpressed with wild-type ARF1, but not with mutant ARF1.** (J)** ALT) and AST in mice serum. **(K)** Schematic diagram illustrating that azelastine binds to ARF1 to inhibit mitochondrial fission and suppress colon tumorigenesis. Bars, SD; *, *P* < 0.05; **, *P* < 0.01; ***, *P* < 0.001.

Next, we determined the effect of azelastine on IQGAP1-ERK signaling. Immunoprecipitation data showed that IQGAP1 interactions with ARF1 and ERK were blocked by azelastine (Figure 6A), suggesting a decrease in ARF1 activity. The ARF1 activation assay showed a significantly lower expression of ARF1-GTP, the active form of ARF1, not only in cells (Figure 6B), but also in the tumor xenografts treated with azelastine (Figure S9A), indicating that azelastine inhibited the activity of ARF1. Besides, we analyzed the expression level of ARF6-GTP, the active form of ARF6, in cells treated with azelastine. As shown in Figure S8, azelastine did not regulate ARF1-GTP expression, illustrating that ARF1, but not ARF6, mediates azelastine's anticancer effect.

Molecular docking performed to explore the potential binding sites of azelastine in ARF1 (Figure 6C) predicted Cys-159 of ARF1 to exert an essential role in azelastine binding to ARF1 (Figure 6D). For validation, we mutated Gys-159 to Gly in ARF1, constructed ARF1-T48S-expressing and ARF1-C159G-expressing plasmids, and overexpressed them and wild-type ARF1 in the ARF1-deficient HT29 and DLD1 cells (Figure 6E). Western blotting showed that the ERK pathway was significantly re-activated in the cells overexpressing either wild-type ARF1 or ARF1-C159G, but not ARF1-T48S (Figure 6F), implying that Thr-48 but not Gys-159 as theARF1 binding site for azelastine.

The repression sensitivity to azelastine in ARF1-deficient CRC cells was markedly restored when either wild-type ARF1 or ARF1-C159G were re-overexpressed to a level comparable to parental cells (Figure 6G). To confirm the Thr-48 binding site in ARF1 for azelastine, the wild-type ARF1 protein and ARF1-T48S mutant protein were purified from cells for binding titration with azelastine *in vitro*. The results from the Biacore assay showed that ARF1 bound to azelastine with the binding constant of Kd = 1.74 × 10^-9^, which was disrupted when Thr-48 of ARF1 was mutated (Kd = 5.94 × 10^-7^) (Figure 6H and Figure S9B-C).

The important role of ARF1 and its Thr-48 binding site in anticancer activity of azelastine was further evaluated *in vivo*. The wild-type ARF1 or ARF1-T48S mutant was overexpressed in ARF1-deficient HT29 and DLD1 cells, which were subcutaneously injected into flanks of nude mice to establish tumor xenografts, and then azelastine was administered in the tumor-bearing mice. Azelastine could not suppress the ability of ARF1-knockout cells to form tumors; however, the antitumor effect of azelastine was restored in cells re-overexpressing wild-type ARF1, but not in the cells with ARF1-T48S re-overexpression (Figure 6I). There was no obvious change in serum levels of ALT and AST in mice, suggesting that azelastine did not cause toxicity (Figure 6J). Taken together, Thr-48 of ARF1 is the azelastine-binding site required for the anticancer bioactivity of azelastine.

## Discussion

Our study explored the repurposing of azelastine, a current anti-allergic drug, as a novel inhibitor of mitochondrial fission to suppress colon cancer. As illustrated in Figure 6K, we provided *in vitro* and *in vivo* evidence to demonstrate that azelastine binds to Thr-48 of ARF1 to block ARF1 activity. The interaction between ARF1-(IQ) IQGAP1 inactivates IQGAP1-mediated ERK signaling, inhibiting mitochondrial fission by regulating p-Drp1 expression to suppress colon tumorigenesis. Thus, azelastine has great potential for further development as a new therapeutic strategy for CRC, providing a successful example of drug repurposing.

The dynamic organelles mitochondria maintain their morphology, length, size, and number through continuous fission and fusion, and abnormal processes, such as increased mitochondrial fission and decreased mitochondrial fusion, cause mitochondrial fragmentation, resistance to apoptosis, and unfavorable tumor progression 33. The mitochondrial fission and fusion are affected by several key molecules, among which mitochondrial fission marker dynamin-related protein 1 (Drp1) is most important 34. It has been documented that overexpression of Drp1 could induce mitochondrial fission and promote cell survival in liver cancer 14, 35, 36, and inhibition of Drp1 activity significantly suppressed cancer cell growth and metastasis 37. However, the mechanism underlying Drp1 regulation remains to be elucidated, which would be crucial for the development of drugs targeting mitochondrial fission. Recent studies reported that the ERK pathway is involved in colon tumorigenesis 38, 39 and is crucial for the phosphorylation of Drp1 at S616, promoting mitochondrial fission and cancer development 34. In this context, our current results demonstrated that azelastine could inhibit Drp1 phosphorylation and mitochondrial fission by inactivating ERK signaling (Figure 2). These data suggested the use of azelastine as a mitochondrial fission-targeting agent.

Interestingly, our study demonstrated that azelastine directly blocks ARF1 activity to inhibit mitochondrial fission. ARF1, a member of the ADP-ribosylation factors family, is a highly conserved Ras family GTPase with inactive (GDP bound) and active (GTP bound) conformations. In most cases, ARF1 acts as a key regulator in maintaining the structure, morphology, and function of Golgi and partitioning during mitosis 40. High expression of ARF1 in a variety of malignant tumors is associated with tumor progression and metastasis. It has been reported that ARF1 may activate the ERK pathway in breast cancer 19 and induce cell adhesion-mediated drug resistance by activating AKT and ERK signaling in multiple myeloma cells 17. Emerging evidence supports that ARF1 can be used as an effective therapeutic target for cancer treatment 17, 41, 42, corroborating our current finding. Indeed, upregulated expression of ARF1 in CRC was negatively correlated with patient survival (Figure 4 and Figure S7). A series of functional experiments demonstrated that knockdown of ARF1 could inhibit mitochondrial fission and suppress CRC cell growth *in vitro* and* in vivo* by inactivating ERK signaling (Figure 3 and Figure S6). More importantly, ARF1 exerted its function by interacting with the IQ domain of IQGAP1 (Figure 5), a member of the evolutionarily conserved IQ-domain GTPase-activating protein family. Thus, our results provided a deeper molecular understanding of ARF1 functionality.

Many studies have documented that IQGAP1 could activate the MAPK pathway by binding to its crucial components, such as MEK and ERK, to promote cancer cell proliferation 43, 44. However, the upstream mechanism in the regulation of IQGAP1 remains unclear. In this context, we demonstrated that ARF1 bound to the IQ domain of IQGAP1 and enhanced its interaction with MEK and ERK. Overexpression of ARF1 failed to increase p-ERK expression and cell proliferation in IQGAP1-deficient cancer cells, suggesting that IQGAP1 was essential for the biological function of ARF1 in cancer (Figure 5). Our findings thus illustrated that ARF1 could serve as a valuable target for CRC intervention.

Our study also revealed that ARF1 is the direct target of azelastine, a phthalazinone derivative, which is an effective drug in treating allergic rhinitis 45, but its effect on cancer has not been reported. The repurposing of non-cancer drugs for cancer treatment is an important strategy to screen drugs with good efficacy and safety. We demonstrated that azelastine exerted profound anti-CRC activity by affecting the ARF1-IQGAP1-ERK-Drp1-mitochondrial fission pathway (Figure 6). Furthermore, we determined Thr-48 in ARF1 as a critical amino acid for azelastine-ARF1 interaction. Histological examination of the liver, kidney, and lung revealed that azelastine treatment had no significant toxic effects (Figure 1D). The suggested maximum oral dose of azelastine as an anti-allergic drug for children is 4 mg/day, which is similar to the dosage used in our animal experiments (10 mg/kg every two days). The significant anticancer effect and its mechanism of action in CRC may position azelastine as an excellent drug repurposing candidate.

Thus, our study illustrated that high ARF1 expression was correlated with poor prognosis of CRC patients, and its interaction with IQGAP1 induced activation of the ERK pathway to promote colon tumorigenesis. Anti-allergic drug azelastine could directly bind to and inactivate ARF1 to block the IQGAP1-mediated ERK signaling, thus inhibiting mitochondrial fission to suppress CRC proliferation *in vitro* and *in vivo*. These findings identified ARF1 as a useful cancer biomarker, and support the potential of azelastine as a novel therapeutic drug for the treatment of colorectal cancer.

## Supplementary Material

Supplementary figures and tables.

## Figures and Tables

**Table 1:** Correlation between ARF1 expression levels and clinicopathological parameters in 202 cases of colorectal cancer

Variable	n	Low ARF1	High ARF1	*P* value
**Age (years)**				
≤55	26	12	14	
>55	176	112	64	0.087
**Gender**				
Female	96	58	38	
Male	106	66	40	0.787
**T-Stage**				
1/2	12	11	1	
3/4	190	113	77	0.026*
**N-Stage**				
N0	122	82	40	
N1	80	42	38	0.035*
**M-Stage**				
M0	198	121	77	
M1	4	3	1	0.572
**Grade**				
I & II	136	84	52	
III & IV	66	40	26	0.873

## Abbreviations

CRC: colorectal cancer
FDA: Food and Drug Administration
Co-IP: Co-immunoprecipitation
ARF1: ADP-ribosylation factor 1
IQGAP1: IQ-domain GTPase-activating protein 1
DIA: data-independent acquisition
DARTS: Drug Affinity Responsive Target Stability
Drp1: Dynamin related protein 1
TCGA: The Cancer Genome Atlas
GEO: Gene Expression Omnibus
IPA: Ingenuity Pathway Analysis
IPTG: isopropyl β-D-thiogalactopyranoside
PBS: phosphate-buffered saline
PVDF: polyvinylidene fluoride
TBST: Tris-buffered saline-Tween
PLGEM: power law global error model
DMSO: dimethyl sulfoxide
RGCT: Ras-GAP C-terminus domain
GRD: Ras-GAP domain
IQ: Isoleucine/glutamine-containing
WW: WW domain
CC: coiled-coil
CH: calponin homology
ALT: Alanine aminotransferase
AST: aspartate aminotransferase
TEM: Transmission electron microscope
TUNEL: TdT-mediated dUTP nick-end labeling
