# Supplementary material for: Anti-allergic drug azelastine suppresses colon tumorigenesis by directly targeting ARF1 to inhibit IQGAP1-ERK-Drp1-mediated mitochondrial fission
Source: Theranostics. 2021 Jan 1;11(4):1828–44. doi: 10.7150/thno.48698 (PMC7778598; doi:10.7150/thno.48698)
Supplement: Supplementary file 1 — Supplementary figures and tables. [file thnov11p1828s1.pdf]

Supplementary Figure Legends

Figure S1. Azelastine inhibits the proliferation of CRC cells with no toxic effects in mice. (A) Comparison of colony formation ability of HT29, DLD1 and HCT116 cells treated with indicated concentrations of azelastine. **(B)** Soft agar colony formation. **(C)** Body weight of nude mice. **(D)** Representative images of the liver, kidney and lung tissues stained with hematoxylin and eosin (H & E). Bars, SD; *, $P < 0.05$; **, $P < 0.001$.

Figure S2. Analysis of differentially expressed proteins in azelastine-treated HT29 cells by PLGEM. (A) PLGEM fitting of the abundance of azelastine-regulated proteins. **(B)** Q–Q plot of the residual versus standard normal. **(C)** Residual distribution along with the rank of mean abundances. **(D)** Histogram of residuals of identified proteins.

Figure S3. Azelastine induces mitochondrial dysfunction in CRC cells. Western blotting was performed to determine the expression of Bcl-xL, Bax, and Bcl-2 in HT29 and DLD1 cells treated with indicated concentrations of azelastine for 48 h.

Figure S4. Activation of the ERK pathway significantly abrogates the effect of azelastine on mitochondria. (A) Immunofluorescent staining was carried out with MitoTracker® probe to compare the morphology of mitochondria in the azelastine-treated HT29 and DLD1 cells. **(B)** p-ERK expression was detected by Western blotting. **(C-F)** CRC cells overexpressing MEK were treated with indicated concentrations of azelastine for 48 h. WST-1, immunofluorescent staining, and Western blotting were performed to compare cell viability **(D),** morphology of mitochondria **(E)**, and p-Drp1 expression **(F)**. **(G)** Comparison of anti-proliferation ability of azelastine and Rotenone

in HT29 and DLD1 cells. Bars, SD; *, $P < 0.05$; **, $P < 0.01$.

Figure S5. ARF1, but not HRH1, mediates the anticancer effect of azelastine. (A) HRH1-deficient CRC cell lines were established. **(B)** WST-1 assay was used to measure cell viability in HRH1-deficient CRC cell lines treated with various concentrations of azelastine. **(C)** Experimental schema of DARTS technology to identify the direct target of azelastine. **(D)** ARF1-deficient CRC cell lines were established. **(E)** WST-1 assay was used to measure cell viability in ARF1-deficient HT29 and DLD1cells treated with various concentrations of azelastine. **(F)** HT29 and DLD1 cells were treated with Mdivi-1 (10μM) for 6 h, then treated with azelastine (20μM); WST-1 assay was used to compare the cell viability. Bars, SD; *, $P < 0.05$; **, $P < 0.01$.

Figure S6. ARF1 exerts its oncogenic function through ERK signaling. (A) Successful establishment of ARF1-overexpressing HCT116 and RKO cells. **(B)** WST-1 assay was performed to determine cell proliferation rate in ARF1-overexpressing CRC cells and control cells. **(C)** The abilities of ARF1-overexpressing HCT116 and RKO cells as well as control cells to form colonies were examined. **(D)** Comparison of anchorage-independent growth ability by soft agar assay. **(E-H)** Comparison of p-ERK expression **(E)**, cell proliferation rate **(H)**, anchorage-dependent growth ability **(F)** and anchorage-independent growth ability **(G)** in ARF1-overexpressing CRC cells in the presence or absence of U0126 (5 μM), and the control cells. **(I)** Successful establishment of inducible knockdown of ARF1 in HT29 and DLD1 cells. **(J-L)** Effect of ARF1 knockdown on proliferation **(J)** and anchorage-independent growth of HT29 and DLD1 cells **(K)**. **(L)** Comparison of the morphology of mitochondria in ARF1-knockdown HT29 and DLD1 cells and control cells. Bars, SD; *, $P < 0.05$. Bars, SD;

*, $P < 0.05$; **, $P < 0.01$; ***, $P < 0.001$.

Figure S7. Clinical significance of ARF1. (A) Comparison of ARF1 expression in 20 pairs of CRC tumors and adjacent normal tissues. **(B)** Data from starBase v3.0 showed that the expression of ARF1 was higher in various cancers than normal tissues. **(C)** Data from TCGA and GEO databases indicated that ARF1 expression was correlated with poor survival in various cancers. Bars, SD; *, $P < 0.05$; **, $P < 0.01$; ***, $P < 0.001$.

Figure S8. Azelastine does not regulate ARF6 expression or activity in CRC cells. The expression and activity of ARF6 in HT29 and DLD1 cells treated with different concentrations of azelastine for 48 h were measured.

Figure S9. Azelastine directly binds to ARF1. (A) Comparison of ARF1 expression and activity in the azelastine-treated tumor xenografts. **(B)** Biacore assay showed that ARF1 binds to azelastine with a Kd of 1.74×10^{-9}. **(C)** Biacore assay showed that ARF1-T48S mutant binds to azelastine with a significantly increased Kd of 5.94×10^{-7}.

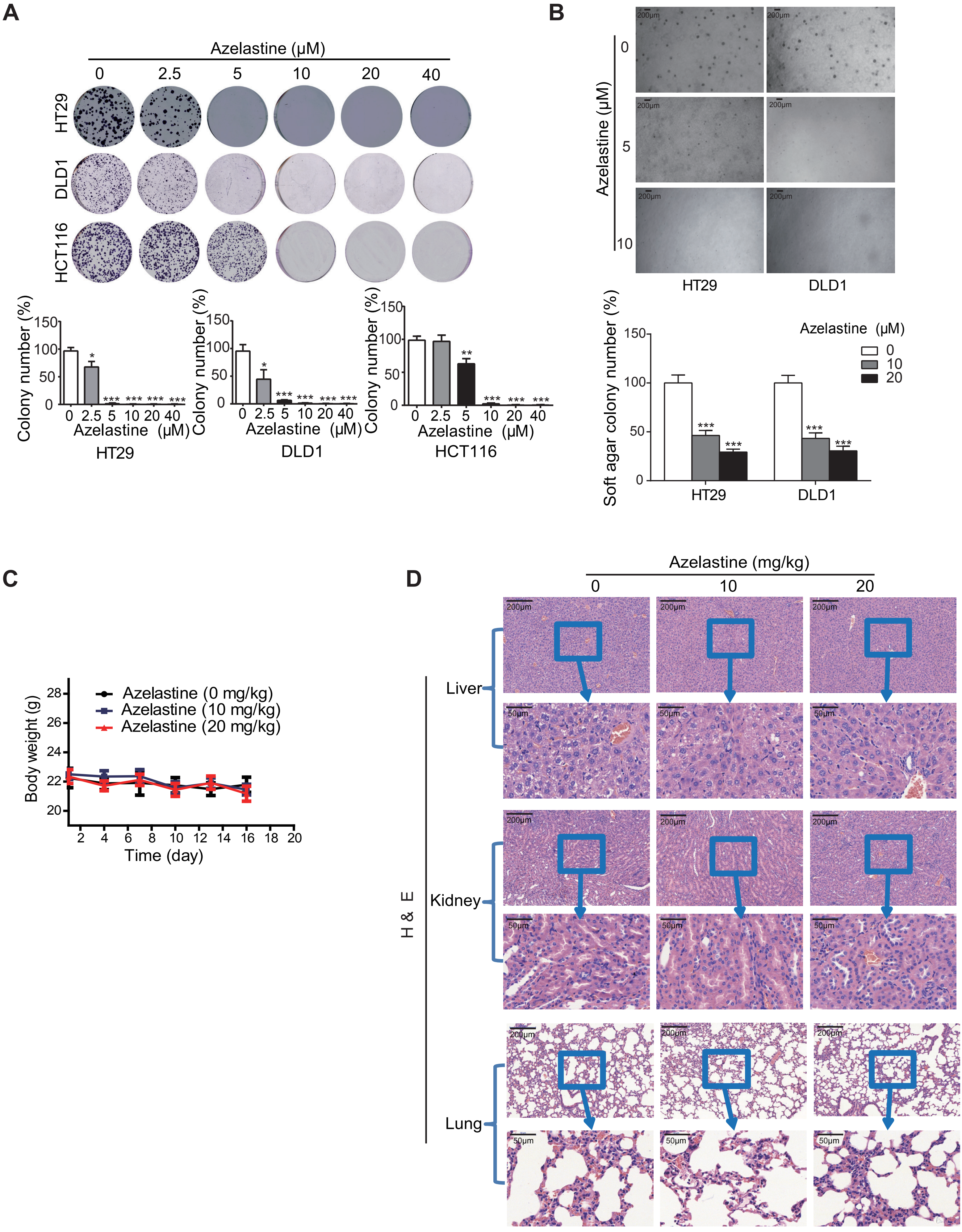
A
Azelastine (μM)
0 2.5 5 10 20 40
HT29
DLD1
HCT116
Colony number (%)
Azelastine (μM)
HT29
DLD1
HCT116
B
Azelastine (μM)
0
5
10
200μm
HT29
DLD1
Soft agar colony number (%)
Azelastine (μM)
0
10
20
C
Body weight (g)
Time (day)
Azelastine (0 mg/kg)
Azelastine (10 mg/kg)
Azelastine (20 mg/kg)
D
Azelastine (mg/kg)
0
10
20
H & E
Liver
Kidney
Lung
200μm
50μm

Figure S1

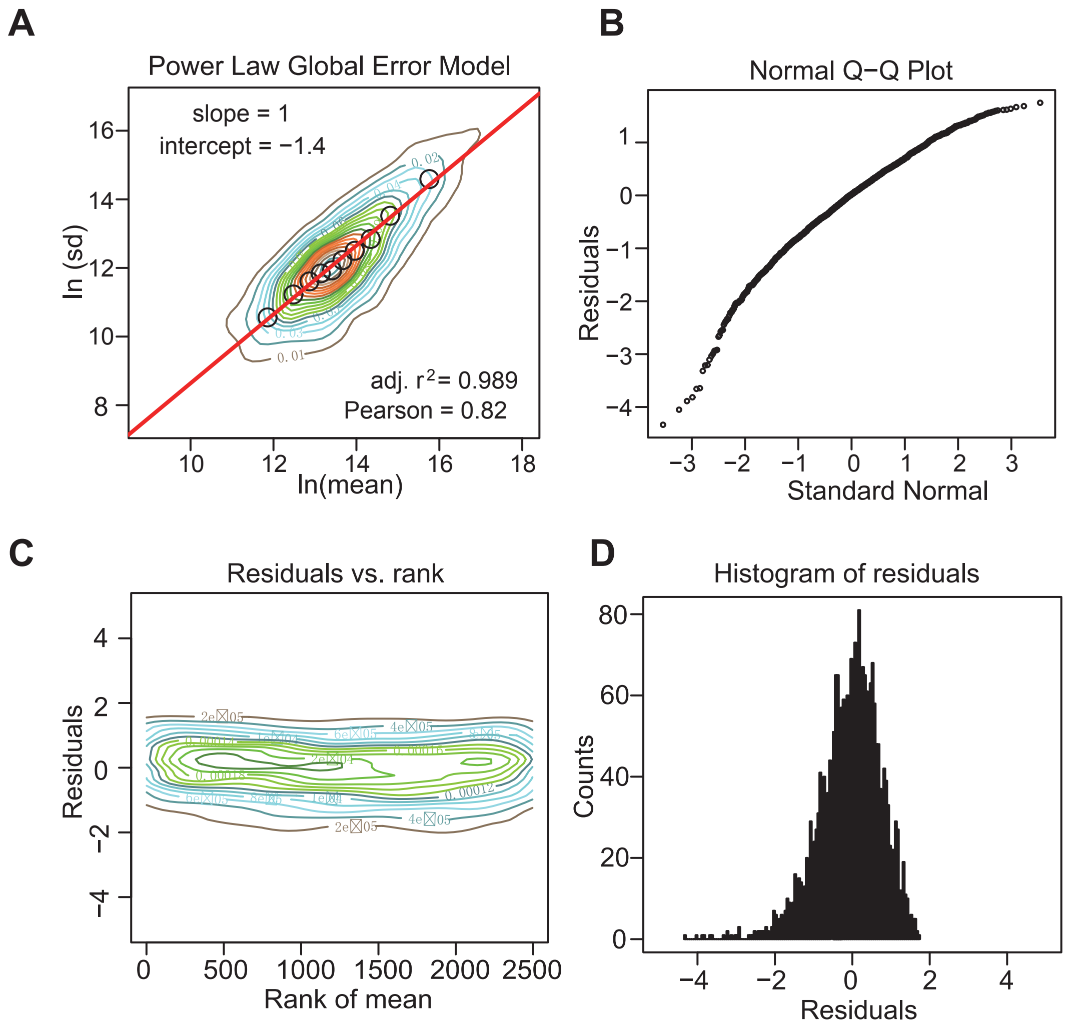
A
Power Law Global Error Model
slope = 1
intercept = −1.4
adj. r2= 0.989
Pearson = 0.82
ln (sd)
ln(mean)
B
Normal Q−Q Plot
Residuals
Standard Normal
C
Residuals vs. rank
Residuals
Rank of mean
D
Histogram of residuals
Counts
Residuals

Figure S2

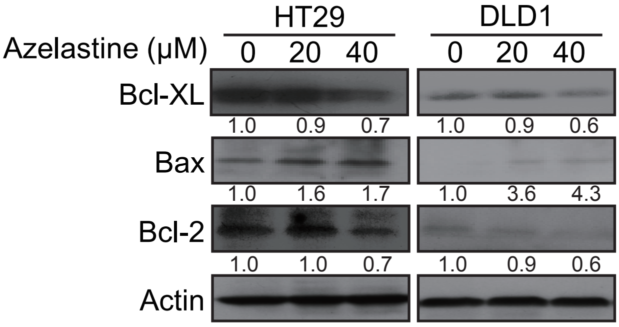
HT29
DLD1
Azelastine (μM)
0 20 40
0 20 40
Bcl-XL
1.0 0.9 0.7
1.0 0.9 0.6
Bax
1.0 1.6 1.7
1.0 3.6 4.3
Bcl-2
1.0 1.0 0.7
1.0 0.9 0.6
Actin

Figure S3

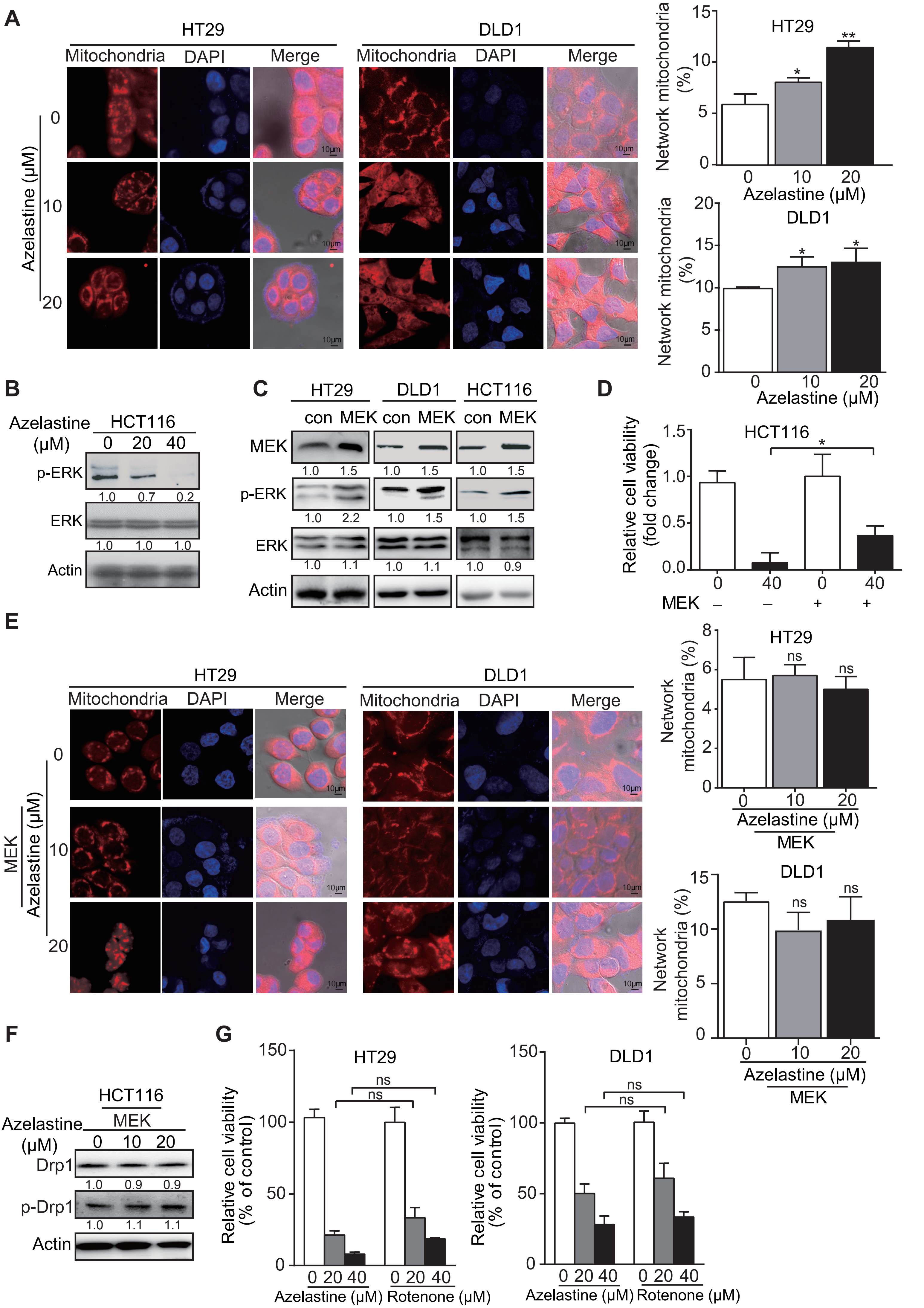
A
HT29
DLD1
Mitochondria
DAPI
Merge
Azelastine (μM)
0
10
20
10μm
Network mitochondria (%)
Azelastine (μM)
B
Azelastine (μM)
HCT116
0 20 40
p-ERK
1.0 0.7 0.2
ERK
1.0 1.0 1.0
Actin
C
HT29
DLD1
HCT116
con MEK con MEK con MEK
MEK
1.0 1.5 1.0 1.5 1.0 1.5
p-ERK
1.0 2.2 1.0 1.5 1.0 1.5
ERK
1.0 1.1 1.0 1.1 1.0 0.9
Actin
D
HCT116
Relative cell viability (fold change)
MEK – – + +
E
MEK
ns
F
HCT116
MEK
Azelastine (μM)
0 10 20
Drp1
1.0 0.9 0.9
p-Drp1
1.0 1.1 1.1
Actin
G
Relative cell viability (% of control)
Azelastine (μM)
Rotenone (μM)

Figure S4

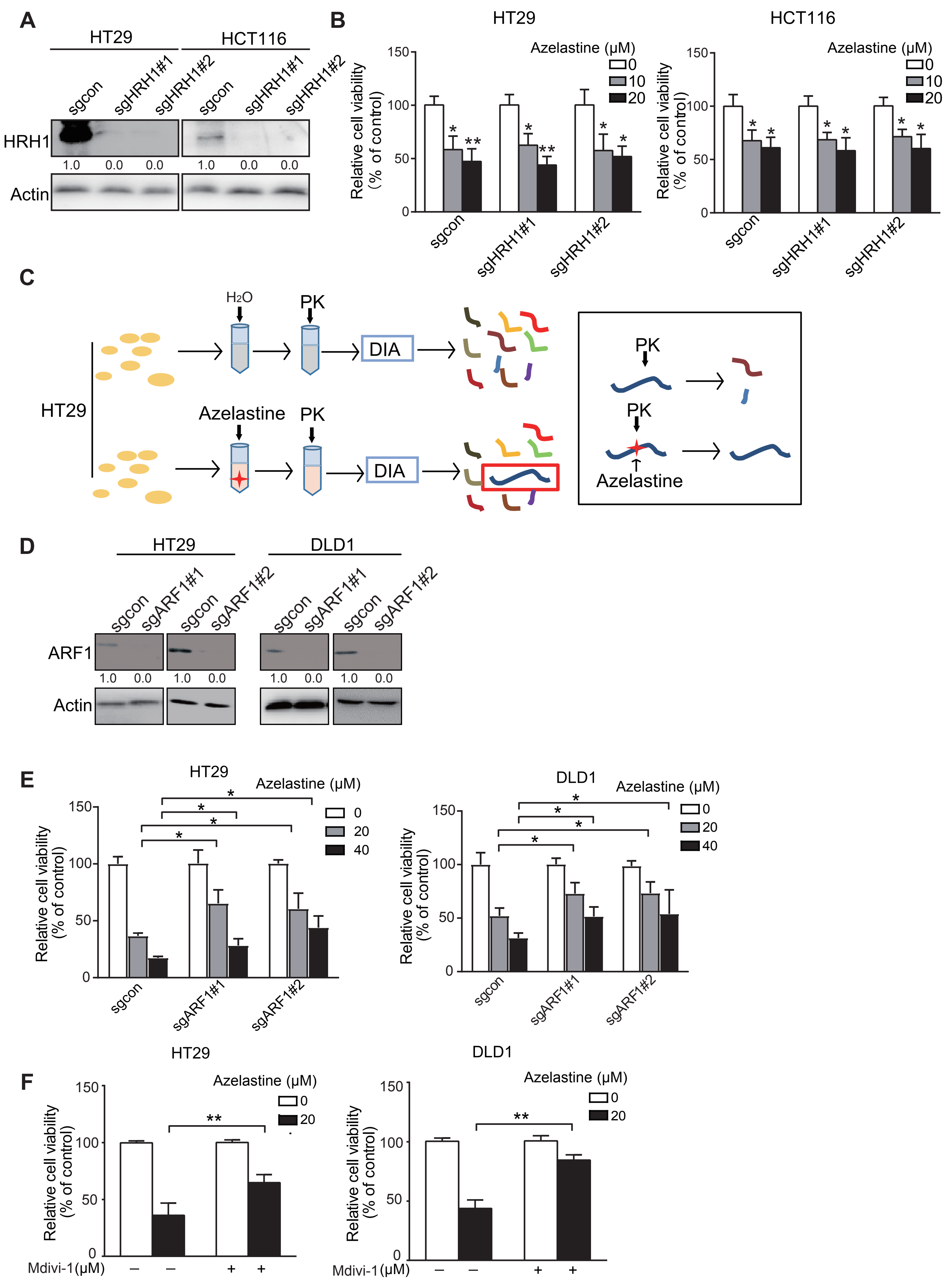
A
HT29
HCT116
sgcon
sgHRH1#1
sgHRH1#2
HRH1
Actin
1.0 0.0 0.0
B
Azelastine (μM)
0
10
20
Relative cell viability (% of control)
C
H2O
PK
DIA
HT29
Azelastine
D
DLD1
sgARF1#1
sgARF1#2
ARF1
E
40
F
Mdivi-1(μM)

Figure S5

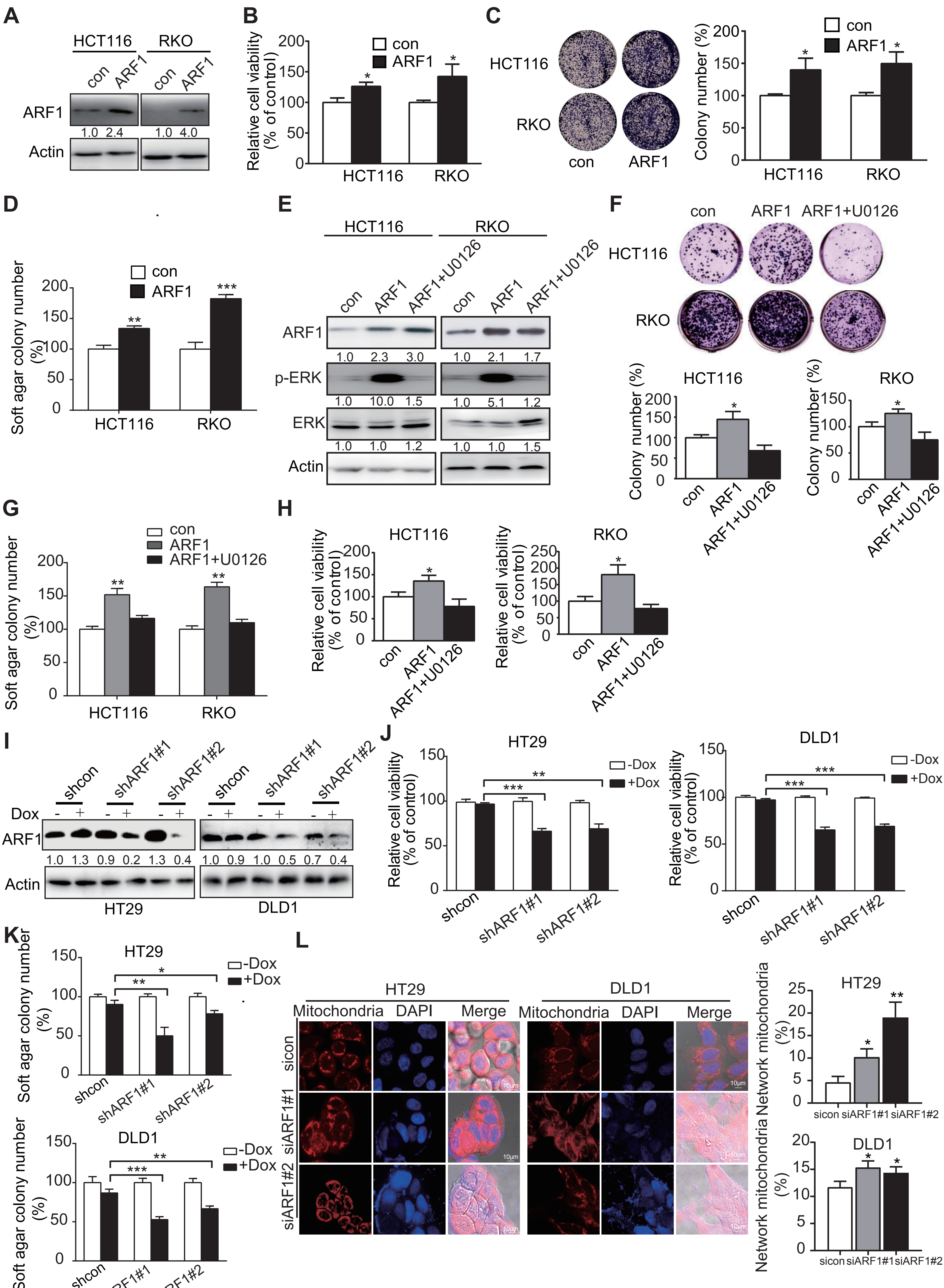
A
HCT116
RKO
con
ARF1
ARF1
1.0 2.4
1.0 4.0
Actin
B
Relative cell viability (% of control)
con
ARF1
HCT116
RKO
C
HCT116
RKO
con
ARF1
Colony number (%)
D
Soft agar colony number (%)
E
p-ERK
ERK
ARF1+U0126
F
G
H
I
shcon
shARF1#1
shARF1#2
Dox
HT29
DLD1
J
-Dox
+Dox
K
L
Mitochondria
DAPI
Merge
sicon
siARF1#1
siARF1#2
Network mitochondria (%)

Figure S6

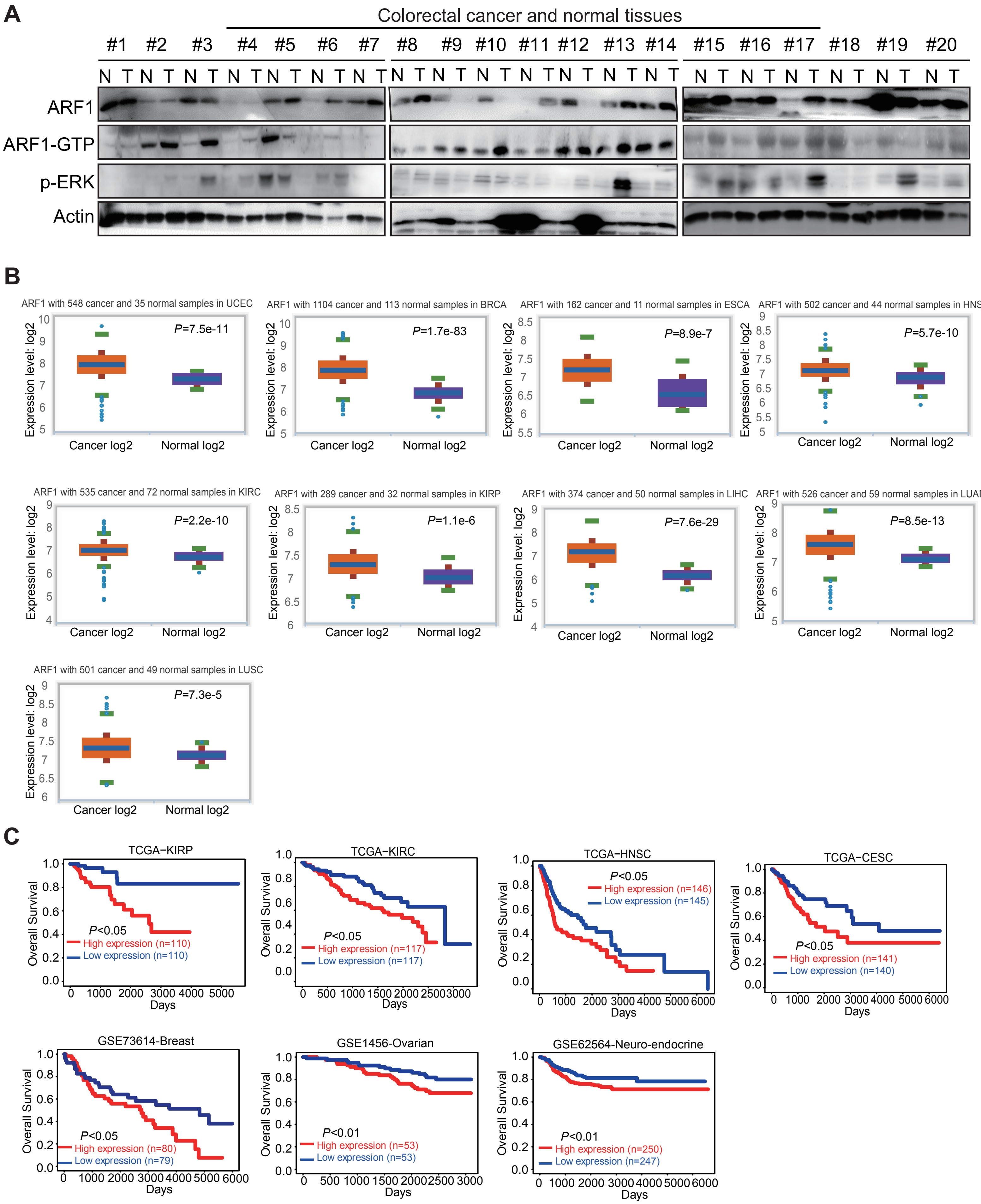
A
Colorectal cancer and normal tissues
#1 #2 #3 #4 #5 #6 #7 #8 #9 #10 #11 #12 #13 #14 #15 #16 #17 #18 #19 #20
N T N T N T N T N T N T N T N T N T N T N T N T N T N T N T N T N T N T N T N T
ARF1
ARF1-GTP
p-ERK
Actin
B
ARF1 with 548 cancer and 35 normal samples in UCEC
P=7.5e-11
ARF1 with 1104 cancer and 113 normal samples in BRCA
P=1.7e-83
ARF1 with 162 cancer and 11 normal samples in ESCA
P=8.9e-7
ARF1 with 502 cancer and 44 normal samples in HNSC
P=5.7e-10
ARF1 with 535 cancer and 72 normal samples in KIRC
P=2.2e-10
ARF1 with 289 cancer and 32 normal samples in KIRP
P=1.1e-6
ARF1 with 374 cancer and 50 normal samples in LIHC
P=7.6e-29
ARF1 with 526 cancer and 59 normal samples in LUAD
P=8.5e-13
ARF1 with 501 cancer and 49 normal samples in LUSC
P=7.3e-5
Expression level: log2
Cancer log2
Normal log2
C
TCGA-KIRP
P<0.05
High expression (n=110)
Low expression (n=110)
TCGA-KIRC
P<0.05
High expression (n=117)
Low expression (n=117)
TCGA-HNSC
P<0.05
High expression (n=146)
Low expression (n=145)
TCGA-CESC
P<0.05
High expression (n=141)
Low expression (n=140)
GSE73614-Breast
P<0.05
High expression (n=80)
Low expression (n=79)
GSE1456-Ovarian
P<0.01
High expression (n=53)
Low expression (n=53)
GSE62564-Neuro-endocrine
P<0.01
High expression (n=250)
Low expression (n=247)
Overall Survival
Days

Figure S7

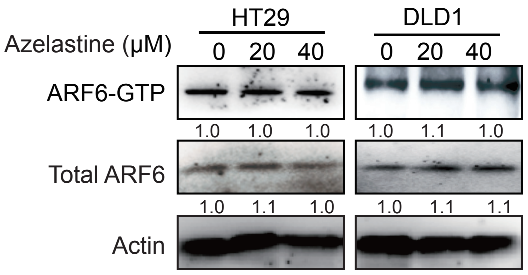
HT29
DLD1
Azelastine (μM)
0 20 40
0 20 40
ARF6-GTP
1.0 1.0 1.0
1.0 1.1 1.0
Total ARF6
1.0 1.1 1.0
1.0 1.1 1.1
Actin

Figure S8

A

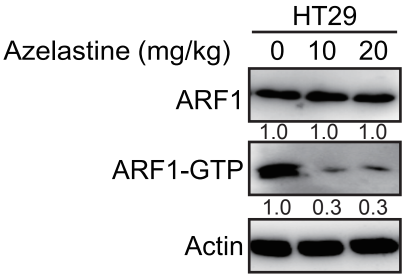

B

ARF1 - PBS medicine

Curve	ka (1/Ms)	kd (1/s)	KD (M)	Rmax (RU)	Conc (M)	tc	Flow (ul/min)	kt (RU/Ms)	RI (RU)	Chi^2 (RU^2)	U-value
	1.19E+07	0.02072	1.74E-09	12.01		5.45E+08				198	95
Cycle: 2 150 µM					1.50E-04		30	1.70E+09	172.6		
Cycle: 3 75 µM					7.50E-05		30	1.70E+09	74.78		
Cycle: 4 37.5 µM					3.75E-05		30	1.70E+09	28.07		
Cycle: 5 18.75 µM					1.88E-05		30	1.70E+09	3.839		
Cycle: 6 9.375 µM					9.38E-06		30	1.70E+09	-10.35		

C

ARF1-T48S - PBS medicine

Curve	ka (1/Ms)	kd (1/s)	KD (M)	Rmax (RU)	Conc (M)	tc	Flow (ul/min)	kt (RU/Ms)	RI (RU)	Chi^2 (RU^2)	U-value
	1.28E+04	0.007602	**5.94E-07**	2.666		1.35E+17				0.443	33
Cycle: 5 150 µM					1.50E-04		30	4.18E+17	14.55		
Cycle: 6 75 µM					7.50E-05		30	4.18E+17	9.02		
Cycle: 7 37.5 µM					3.75E-05		30	4.18E+17	4.057		
Cycle: 8 18.75 µM					1.88E-05		30	4.18E+17	1.526		
Cycle: 9 9.375 µM					9.38E-06		30	4.18E+17	0.7115		

Figure S9

Table S1

The sequences of ARF1 siRNA and primers for cloning and mutation of ARF1- and IQGAP1-expressing plasmids

siRNA sequence

**siARF1#1**	5'-CGAUCCUCUACAAGCUUAATTUUAAGCUUGUAGAGGAUCGTT-3'
**siARF1#2**	5'-GGGUCCGAUUUGCCAUCGATTUCGAUGGCAAAUCGGACCCTT-3'

primers for cloning and mutation

**pET-28b-ARF1**	forward primer 5'-CGCGGATCCTGGACGGTTGGGATTAGCG -3'
	reverse primer 5'- CGCGAATTC GGAGGCAGCTTCTGGCACT -3'
**pET-28b-ARF1^{T48S}**	forward primer 5'- TGCAGGGAAGACCTCGATCCTCTA -3'
	reverse primer 5'- CTTCCCTGCAGCATCCAGGCCC -3'
**pcDNA3.1-ARF1^{T48S}**	forward primer 5- GCTGCAGGGAAGACCTCGATCCTCTA-3'
	reverse primer 5'- TGGTCTTCCCTGCAGCATCCAGGCCC -3'
**pcDNA3.1-ARF1^{G159C}**	forward primer 5'-GTACATTCAGGCCACCGGCGCCACCAGC-3'
	reverse primer 5'- CGGTGGCCTGAATGTACCAGTTCCTGT-3'
**pcDNA3.1-IQGAP1-ΔRGCT-flag**	forward primer 5'-CGCGGATCCATGTCCGCCGCAGACGAG-3'
	reverse primer 5'-CGCCTCGAGTTACTTCCCGTAGAACTTTTTGTTGAGAA-3'
**pcDNA3.1-IQGAP1-ΔRGCT/GRD-flag**	forward primer 5'-TAGTCCAGTGTGGTGGAATTCATGTCCGCCGCAGACGAG-3'
	reverse primer 5'-TGCTGGATATCTGCAGAATTCTTACTTGTCATCGTCGTCCTTGTAG-3'
**pcDNA3.1-IQGAP1-ΔRGCT/GRD/IQ-flag**	forward primer 5'-TAGTCCAGTGTGGTGGAATTCATGTCCGCCGCAGACGAG-3'
	reverse primer 5'-TGCTGGATATCTGCAGAATTCTTACTTGTCATCGTCGTCCTTGTAGTCAGGTTCATCCCAT-3'

Table S2
The list of FDA-approved drugs with the most significantly inhibitory effect in HT29 cells

FDA-approved drugs	Inhibitor ratio (%)
Motolimod (VTX-2337)	100
Vitamin E	100
Rivastigmine	100
Cilengitide	100
Marimastat (BB-2516)	100
Mepiroxol	100
D panthenol	100
Benzyl alcohol	100
Nialamide	100
Ethosuximide	99.2
EPZ-6438	99.1
Hexylresorcinol	97.9
Ledipasvir (GS5885)	97.9
Oxytetracycline Dihydrate	97.7
Tranylcypromine (2-PCPA) HCl	97.0
Micafungin Sodium	97.0
Pimecrolimus	96.9
Dexamethasone Acetate	96.4
Artemisinin	96.2
Batimastat (BB-94)	95.3
Fludarabine Phosphate	94.6
Edaravone	94.6
Amprenavir	94.3
DL-Panthenol	94.1
Epacadostat (INCB024360)	94.0
Chloroxylenol	93.8
Doxapram HCl	92.9
Salmeterol Xinafoate	92.7
Mitoxantrone HCl	92.6
Clofibric Acid	91.7
Dienogest	91.5
Clomifene citrate	91.2
Maprotiline HCl	90.8
Clofoctol	90.7
Fenbufen	90.7
Phenazopyridine HCl	90.3
Sulfisoxazole	90.1
Rufinamide	90.0

Acarbose	90.0
Azelastine HCl	90.0
Pidotimod	89.4
Loperamide HCl	89.1
Dapson	89.1
Serotonin HCl	88.9
Loxoprofen	88.9
Piromidic Acid	88.7
Etodolac	88.5
Fexofenadine HCl	88.0
Mecarbinate	87.8
Apatinib	87.8
Oseltamivir Phosphate	87.8
Arecoline	87.8
Cefotaxime sodium	87.6
Atovaquone	87.6
Mupirocin	87.5
L-Thyroxine	86.3
Clorsulon	86.3
Clindamycin	86.3
Bufexamac	86.3
Uprosertib (GSK2141795)	86.3
Miconazole	86.1
Miconazole Nitrate	85.1
Disulfiram	84.5
Tylosin tartrate	84.4
Itraconazole	84.1
Aripiprazole	83.9
Tofacitinib (CP-690550) Citrate	83.8
Tivantinib (ARQ 197)	83.5
Trifluridine	83.1
Chloroxine	83.1
Dexrazoxane HCl (ICRF-187, ADR-529)	82.3
Amisulpride	82.3
Lapatinib	82.0
Mestranol	82.0
Crenolanib (CP-868596)	82.0
Isosorbide Mononitrate	81.5
Lansoprazole	80.8
Piperazine	80.2

Table S3
The differently expressed proteins in azelastine-treated HT29 cells
(fold change ≥ 1.5)

**Protein Accessions**	**Fold change**	**Protein Accessions**	**Fold change**
P51114	-6.832667548	Q6Y7W6	-1.89311
P98194	-4.881613599	Q96C90	-1.86772
P06493	-4.011846735	Q9H2J4	-1.85013
P49959	-3.778330327	O60826	-1.84335
Q96EE4	-3.713010603	Q9H6Z4	-1.82366
Q9UPY5	-3.594322012	O43432	-1.81861
Q8IWV7	-3.547035429	Q9NRX1	-1.80696
Q7LGA3	-3.411066361	Q969X5	-1.80336
Q9UNF1	-2.657584065	Q9GZP4	-1.8026
Q86TI2	-2.620597653	Q06546	-1.78926
Q13158	-2.529382974	Q6ZXV5	-1.78211
P49736	-2.518350892	Q16850	-1.78099
Q9GZP8	-2.448243532	O00515	-1.77628
Q9UKV3	-2.421533349	P56545	-1.75239
P49023	-2.421242407	O14896	-1.74558
Q8NBM4	-2.391938849	Q9Y2Q5	-1.74329
Q9H1I8	-2.351915695	Q9BPX3	-1.74037
O60832	-2.290401083	Q92643	1.75071
P62857	-2.256702318	P23368	1.753112
O75794	-2.238938717	Q6P582	1.756873
P62829	-2.222693971	Q7Z4Q2	1.764169
Q9UPT8	-2.126620775	O96000	1.767746
P52292	-2.112608561	O95168	1.767765
O95793	-2.102988027	P13646	1.768566
O75909	-2.093797206	P32320	1.769238
Q11201	-2.092881663	Q96L92	1.770828
Q14554	-2.085752411	Q9H330	1.775943
Q9UBB6	-2.083485385	Q7KZI7	1.780843
Q96K17	-2.072919845	Q9NTI5	1.781345
O14737	-2.06585936	Q9NXV6	1.787348
Q9NPI6	-2.026963436	Q9UHL4	1.789826
Q9GZZ9	-2.004030245	Q9Y6M9	1.791888
Q8WVV4	-1.984775534	P68871	1.7925
Q12849	-1.980742408	Q9Y6C9	1.792512
Q96FZ2	-1.94034872	Q13620	1.792576
Q92692	-1.934335601	Q9GZN4	1.798262
Q5JSH3	-1.928809391	A1L0T0	1.799078
Q9C0H2	-1.912084516	O75348	1.801763

Q13190	-1.906999226	P07910	1.823556
P31930	1.825469952	Q9Y639	2.125127
P80188	1.830112256	P13164	2.147616
P16930	1.831148084	P08727	2.154317
Q9H1P3	1.835795556	O00567	2.191989
Q96N66	1.836272058	P16403	2.1951
Q7Z3B4	1.838581691	P62736	2.201723
Q9BPW8	1.842549825	Q8IXQ6	2.201941
P23142	1.84634062	Q92974	2.207565
Q93050	1.846548064	Q9NVH1	2.216658
Q9BVT8	1.867339359	Q9NV70	2.24926
Q03405	1.876755196	P15313	2.257335
P60059	1.878136991	P63279	2.268476
Q9GZQ8	1.887993316	O60613	2.312953
P48729	1.900969025	P21796	2.326782
Q9P0J0	1.901341864	Q5VTL8	2.327317
P54709	1.905999991	Q8N4J0	2.422933
P05165	1.911535858	Q9Y277	2.471473
Q9UIJ7	1.911776593	Q9H061	2.494181
P05787	1.915345603	P45880	2.523518
Q99623	1.916920168	P35900	2.538734
P14854	1.918578906	Q6UXN9	2.574194
Q15428	1.924644885	P14927	2.614551
Q92621	1.927851009	P30046	2.628983
Q04695	1.934041174	P04004	2.705816
Q99988	1.938474044	Q14978	2.723349
P60520	1.95314899	Q9UI26	2.766125
O96008	1.956607141	Q9Y3D9	2.806933
Q14254	1.963667206	P26447	3.061833
O43809	1.965736593	Q8IWB7	3.282603
Q14739	1.967841598	Q8N684	3.319223
Q99828	1.982049036	P16401	3.445445
Q9NP81	1.985789514	Q96QV6	3.520274
P61421	1.987312978	O75367	3.555367
P54886	2.003287332	Q12996	3.649475
P26358	2.005555685	P62807	3.987542
P24557	2.022355756	P62805	4.083643
P02538	2.036050801	Q71DI3	4.252389
Q12888	2.050255181	P33778	4.302705
Q96AB3	2.056791308	P04908	4.467697
Q9Y2X3	2.065400954	P84243	4.639078
Q92673	2.069227725	P68431	4.787733
P02647	2.082396233	Q71UI9	5.200663

O94855	2.085935354	Q9BTY7	9.535954

Table S4
The list of 1056 FDA-approved drugs

Number	Product Name	CAS Number	Number	Product Name	CAS Number
1	Nelarabine	121032-29-9	26	Prasugrel	150322-43-3
2	Bleomycin Sulfate	9041-93-4	27	Ramelteon	196597-26-9
3	Clofarabine	123318-82-1	28	Cinacalcet HCl	364782-34-3
4	Dacarbazine	4342-03-4	29	Celecoxib	169590-42-5
5	Dexrazoxane HCl (ICRF-187, ADR-529)	149003-01-0	30	Avagacestat (BMS-708163)	1146699-66-2
6	Epirubicin HCl	56390-09-1	31	Vemurafenib (PLX4032, RG7204)	918504-65-1
7	Oxaliplatin	61825-94-3	32	Acarbose	56180-94-0
8	Etoposide	33419-42-0	33	Adapalene	106685-40-9
9	Raloxifene HCl	82640-04-8	34	Altretamine	645-05-6
10	Fludarabine Phosphate	75607-67-9	35	Amisulpride	71675-85-9
11	Flavopiridol (Alvocidib)	146426-40-6	36	Aniracetam	72432-10-1
12	Topotecan HCl	119413-54-6	37	Artemisinin	63968-64-9
13	Letrozole	112809-51-5	38	Asenapine	65576-45-6, 135883-08-8
14	Temozolomide	85622-93-1	39	Benazepril HCl	86541-74-4
15	Tamoxifen	10540-29-1	40	Budesonide	51333-22-3
16	Vincristine	2068-78-2	41	Bumetanide	28395-03-1
17	Agomelatine	138112-76-2	42	Carmofur	61422-45-5
18	Amuvatinib (MP-470)	850879-09-3	43	Cetirizine DiHCl	83881-52-1
19	Leflunomide	75706-12-6	44	Cilnidipine	132203-70-4
20	Enzalutamide (MDV3100)	915087-33-1	45	Cilostazol	73963-72-1
21	Dienogest	65928-58-7	46	Floxuridine	50-91-9
22	Entecavir Hydrate	209216-23-9	47	FT-207 (NSC 148958)	17902-23-7
23	Nepafenac	78281-72-8	48	Ifosfamide	3778-73-2
24	Rufinamide	106308-44-5	49	Megestrol Acetate	595-33-5
25	Posaconazole	171228-49-2	50	Mercaptopurine (6-MP)	50-44-2

51	Streptozotocin (STZ)	18883-66-4	76	Acitretin	55079-83-9
52	Dexamethasone (DHAP)	50-02-2	77	Bafetinib (INNO-406)	859212-16-1
53	Doxazosin Mesylate	77883-43-3	78	Doripenem Hydrate	364622-82-2
54	Edaravone	89-25-8	79	Gestodene	60282-87-3
55	Ellagic acid	476-66-4	80	Drospirenone	67392-87-4
56	Etodolac	41340-25-4	81	Ruxolitinib (INCB018424)	941678-49-5
57	Etomidate	33125-97-2	82	Isotretinoin	4759-48-2
58	Felbamate	25451-15-4	83	Lopinavir	192725-17-0
59	Fluconazole	86386-73-4	84	Meropenem	96036-03-2
60	Flumazenil	78755-81-4	85	Mianserin HCl	21535-47-7
61	Fluoxetine HCl	56296-78-7	86	Mosapride Citrate	112885-42-4
62	Fluvoxamine maleate	61718-82-9	87	Nafamostat Mesylate	82956-11-4
63	Gatifloxacin	112811-59-3	88	Omeprazole	73590-58-6
64	Genistein	446-72-0	89	Methimazole	60-56-0
65	Glimepiride	93479-97-1	90	Metolazone	17560-51-9
66	Ivermectin	70288-86-7	91	Cefoperazone	62893-19-0
67	Ketoconazole	65277-42-1	92	Silodosin	160970-54-7
68	Lansoprazole	103577-45-3	93	Riluzole	1744-22-5
69	Levetiracetam	102767-28-2	94	Risperidone	106266-06-2
70	Lidocaine	137-58-6	95	Sulfapyridine	144-83-2
71	Loratadine	79794-75-5	96	Sulfameter	651-06-9
72	Losartan Potassium (DuP 753)	124750-99-8	97	Prilocaine	721-50-6
73	Rigosertib (ON-01910)	1225497-78-8	98	Darunavir Ethanolate	635728-49-3
74	Epothilone B (EPO906, Patupilone)	152044-54-7	99	Theophylline	58-55-9
75	Amonafide	69408-81-7	100	Prednisone	53-03-2

101	Acetylcysteine	616-91-1	126	Tretinoin	302-79-4
102	Alendronate	121268-17-5	127	Phenylbutazone	50-33-9
103	Ethinyl Estradiol	57-63-6	128	Ezetimibe	163222-33-1
104	Naproxen	26159-34-2	129	Enalaprilat Dihydrate	84680-54-6
105	Nitazoxanide	55981-09-4	130	Dofetilide	115256-11-6
106	Triamcinolone Acetonide	76-25-5	131	Isradipine	75695-93-1
107	Orlistat	96829-58-2	132	Estrone	53-16-7
108	Allopurinol	315-30-0	133	Flucytosine	2022-85-7
109	Zafirlukast	107753-78-6	134	Trichlormethiazide	133-67-5
110	Acetaminophen	103-90-2,16958-94-4	135	Loteprednol etabonate	82034-46-6
111	Erythromycin	114-07-8	136	（6-）ε-Aminocaproic acid	60-32-2
112	Amphotericin B	1397-89-3	137	Aminoglutethimide	125-84-8
113	Ibuprofen	15687-27-1	138	Aminophylline	317-34-0
114	Amprenavir	161814-49-9	139	Amorolfine HCl	78613-38-4
115	Albendazole	54965-21-8	140	Chloramphenicol	56-75-7
116	Chlorothiazide	58-94-6	141	Flurbiprofen	51543-39-6
117	Methyldopa	555-30-6	142	Disulfiram	97-77-8
118	Ursodiol	128-13-2	143	Mesalamine	89-57-6
119	Nitrofural	59-87-0	144	Ipratropium Bromide	22254-24-6
120	Ketoprofen	22071-15-4	145	Sulfanilamide	63-74-1
121	Ketorolac	74103-07-4	146	Betamethasone Dipropionate	5593-20-4
122	Adenosine	58-61-7	147	Meprednisone	1247-42-3
123	Zolmitriptan	139264-17-8	148	Betamethasone valerate (Betnovate)	2152-44-5
124	Telbivudine	3424-98-4	149	Praziquantel	55268-74-1
125	Monobenzone	103-16-2	150	Busulfan	55-98-1

151	Carbamazepine	298-46-4	176	Gemfibrozil	25812-30-0
152	Hydrocortisone	50-23-7	177	Indapamide	26807-65-8
153	Torsemide	56211-40-6	178	Mitotane	53-19-0
154	Desonide	638-94-8	179	Methylprednisolone	83-43-2
155	Didanosine	69655-05-6	180	Meloxicam	71125-38-7
156	Divalproex Sodium	76584-70-8	181	Mesna	19767-45-4
157	Emtricitabine	143491-57-0	182	Methocarbamol	532-03-6
158	Progesterone	57-83-0	183	Prednisolone	50-24-8
159	Lamivudine	134678-17-4	184	Telmisartan	144701-48-4
160	Eplerenone	107724-20-9	185	Thiabendazole	148-79-8
161	Hydrochlorothia zide	58-93-5	186	Guaifenesin	93-14-1
162	Estradiol	50-28-2	187	Rifabutin	72559-06-9
163	Deferasirox	201530-41-8	188	Nevirapine	129618-40-2
164	Piroxicam	36322-90-4	189	Esomeprazole Magnesium	161973-10-0
165	Gemcitabine	95058-81-4	190	Nicotinic Acid	59-67-6
166	Glipizide	29094-61-9	191	Nimodipine	66085-59-4
167	Glyburide	10238-21-8	192	Nisoldipine	63675-72-9
168	Fomepizole	7554-65-6	193	Oxybutynin	5633-20-5
169	Adefovir Dipivoxil	142340-99-6	194	Enoxacin	74011-58-8
170	Zalcitabine	7481-89-2	195	Pitavastatin Calcium	147526-32-7
171	Azathioprine	446-86-6	196	Rifapentine	61379-65-5
172	Indomethacin	53-86-1	197	Suprofen	40828-46-4
173	Paliperidone	144598-75-4	198	Pyrazinamide	98-96-4
174	Terbinafine	91161-71-6	199	Quetiapine Fumarate	111974-72-2
175	Levonorgestrel	797-63-7	200	Rifampin	13292-46-1

201	Cefditoren Pivoxil	117467-28-4	226	Chlorpheniramine Maleate	113-92-8
202	Sulfadiazine	68-35-9	227	Fenoprofen Calcium	34597-40-5
203	Chlorprothixene	113-59-7	228	Erdosteine	84611-23-4
204	Oxytetracycline (Terramycin)	79-57-2	229	Betaxolol hydrochloride (Betoptic)	63659-19-8
205	Toremifene Citrate	89778-27-8	230	Proparacaine HCl	5875-06-9
206	Ethionamide	536-33-4	231	Pranlukast	103177-37-3
207	Trifluridine	70-00-8	232	Oxfendazole	53716-50-0
208	Azacitidine	320-67-2	233	Carvedilol	72956-09-3
209	Vidarabine	5536-17-4	234	Atracurium Besylate	64228-81-5
210	Teniposide	29767-20-2	235	Butoconazole nitrate	64872-77-1
211	Stanozolol	10418-03-8	236	Azithromycin	83905-01-5
212	Tetrabenazine (Xenazine)	58-46-8	237	Albendazole Oxide	54029-12-8
213	Rifaximin	80621-81-4	238	Flubendazole	31430-15-6
214	Simvastatin	79902-63-9	239	Chloroxine	773-76-2
215	Ramipril	87333-19-5	240	Lomustine	13010-47-4
216	Fenofibrate	49562-28-9	241	Chenodeoxycholic Acid	474-25-9
217	Ranolazine	95635-55-5	242	Cimetidine	51481-61-9
218	Ranitidine	66357-59-3	243	Clemastine Fumarate	14976-57-9
219	Acadesine	2627-69-2	244	Curcumin	458-37-7
220	Acetylcholine Chloride	60-31-1	245	Bifonazole	60628-96-8
221	Acipimox	51037-30-0	246	Pefloxacin Mesylate	70458-95-6
222	Aciclovir	59277-89-3	247	Metoprolol Tartrate	392-17-7
223	Nifedipine	21829-25-4	248	Diethylstilbestrol	56-53-1
224	Amiloride HCl	2016-88-8	249	Diltiazem HCl	33286-22-5
225	Amlodipine besylate (Norvasc)	111470-99-6	250	Diphenhydramine HCl	147-24-0

251	Dapoxetine HCl	129938-20-1	276	Tropicamide	1508-75-4
252	Valaciclovir HCl	124832-27-5	277	Pregnenolone	145-13-1
253	Ganciclovir	82410-32-0	278	Sulfamethoxazole	723-46-6
254	Roxatidine Acetate HCl	93793-83-0	279	Sulfisoxazole	127-69-5
255	Protionamide	14222-60-7	280	Crystal Violet	548-62-9
256	Idoxuridine	54-42-2	281	Haloperidol	52-86-8
257	Sparfloxacin	110871-86-8	282	Phenindione	83-12-5
258	Felodipine	72509-76-3	283	Alibendol	26750-81-2
259	Deflazacort	14484-47-0	284	Irsogladine	57381-26-7
260	Nizatidine	76963-41-2	285	Triamcinolone	124-94-7
261	Carbidopa	28860-95-9	286	Nystatin (Fungicidin)	1400-61-9
262	Valsartan	137862-53-4	287	Isoniazid	54-85-3
263	Dipyridamole	58-32-2	288	Levofloxacin	100986-85-4
264	Hydroxyurea	127-07-1	289	Enalapril Maleate	76095-16-4
265	Potassium Iodide	7681-11-0	290	Menadione	58-27-5
266	Tropisetron	105826-92-4	291	Methoxsalen	298-81-7
267	Nicotinamide (Vitamin B3)	98-92-0	292	Miconazole Nitrate	22832-87-7
268	Vitamin B12	68-19-9	293	Sulfamethizole	144-82-1
269	Diclofenac Sodium	15307-79-6	294	Sulbactam	68373-14-8
270	Avobenzone	70356-09-1	295	Tolfenamic Acid	13710-19-5
271	Amlodipine	88150-42-9	296	Pranoprofen	52549-17-4
272	Metronidazole	443-48-1	297	Sulphadimethoxine	122-11-2
273	Flutamide	13311-84-7	298	Rimantadine	13392-28-4
274	Fluvastatin Sodium	93957-55-2	299	Primidone	125-33-7
275	Tioconazole	65899-73-2	300	Nefiracetam	77191-36-7

301	Nicorandil	65141-46-0	326	Uridine	58-96-8
302	Tamoxifen Citrate	54965-24-1	327	Flunarizine 2HCl	30484-77-6
303	Meglumine	6284-40-8	328	Fenticonazole Nitrate	73151-29-8
304	Aripiprazole	129722-12-9	329	Rebamipide	90098-04-7
305	Sarafloxacin HCl	91296-87-6	330	Epalrestat	82159-09-9
306	Methscopolamin e	155-41-9	331	Aspartame	22839-47-0
307	Amiodarone HCl	19774-82-4	332	Candesartan Cilexetil	145040-37-5
308	Meclizine 2HCl	1104-22-9	333	Phentolamine Mesylate	65-28-1
309	Mometasone furoate	83919-23-7	334	Nimesulide	51803-78-2
310	Propylthiouracil	51-52-5	335	Dyclonine HCl	536-43-6
311	Fluticasone propionate	80474-14-2	336	Cyproterone Acetate	427-51-0
312	Lacidipine	103890-78-4	337	Memantine HCl	41100-52-1
313	Elvitegravir (GS-9137, JTK-303)	697761-98-1	338	Cyproheptadine HCl	969-33-5
314	Maraviroc	376348-65-1	339	Doxifluridine	3094-09-5
315	Raltegravir (MK-0518)	518048-05-0	340	Pioglitazone HCl	112529-15-4
316	Pyrimethamine	58-14-0	341	Lornoxicam	70374-39-9
317	Sulindac	38194-50-2	342	Captopril	62571-86-2
318	Pramipexole dihydrochloride monohydrate	191217-81-9	343	Oxytetracycline Dihydrate	6153-64-6
319	Mirtazapine	85650-52-8	344	Orphenadrine Citrate	4682-36-4
320	Benidipine HCl	91599-74-5	345	Gimeracil	103766-25-2
321	Formoterol Hemifumarate	43229-80-7	346	Cyclophosphamide Monohydrate	6055-19-2
322	Chlormezanone	80-77-3	347	Tolnaftate	2398-96-1
323	Ketotifen Fumarate	34580-14-8	348	Terazosin HCl	70024-40-7
324	Urapidil HCl	64887-14-5	349	Bromhexine HCl	611-75-6
325	Diclazuril	101831-37-2	350	Lovastatin	75330-75-5

351	Tiopronin	1953-02-2	376	Gabexate Mesylate	56974-61-9
352	Balofloxacin	127294-70-6	377	Rasagiline Mesylate	161735-79-1
353	Lafutidine	118288-08-7	378	Naltrexone HCl	16676-29-2
354	Moxonidine	75438-57-2	379	Levosulpiride	23672-07-3
355	Ozagrel HCl	78712-43-3	380	Flunixin Meglumin	42461-84-7
356	Argatroban	74863-84-6	381	Imidapril HCl	89371-37-9
357	Mecarbinate	15574-49-9	382	Vinpocetine	42971-09-5
358	Rosiglitazone HCl	302543-62-0	383	Lapatinib	231277-92-2
359	Atorvastatin Calcium	134523-03-8	384	Cisatracurium Besylate	96946-42-8
360	Famotidine	76824-35-6	385	Dronedarone HCl	141625-93-6
361	Moexipril HCl	82586-52-5	386	Conivaptan HCl	168626-94-6
362	Clevidipine Butyrate	167221-71-8	387	Ibutilide Fumarate	122647-32-9
363	Cilazapril Monohydrate	92077-78-6	388	Probucol	23288-49-5
364	Adiphenine HCl	50-42-0	389	Licofelone	156897-06-2
365	Duloxetine HCl	136434-34-9	390	Dextrose	50-99-7
366	Trimebutine	39133-31-8	391	Xylose	25990-60-7
367	Ivabradine HCl	148849-67-6	392	Mestranol	72-33-3
368	Rivastigmine Tartrate	129101-54-8	393	Naftopidil	57149-07-2
369	Dexmedetomidine HCl (Precedex)	145108-58-3	394	S- (+)-Rolipram	85416-73-5
370	Betaxolol	659-18-7	395	Bazedoxifene HCl	198480-56-7
371	Detomidine HCl	90038-01-0	396	Atropine	5908-99-6
372	Almotriptan Malate	181183-52-8	397	Roflumilast	162401-32-3
373	Ambrisentan	177036-94-1	398	Neratinib (HKI-272)	698387-09-6
374	Bexarotene	153559-49-0	399	LDE225 (NVP-LDE225,Erismodegib)	956697-53-3
375	Temocapril HCl	110221-44-8	400	Sitafloxacin Hydrate	163253-35-8

401	Dabigatran Etexilate	211915-06-9	426	Orotic acid (6-Carboxyuracil)	65-86-1
402	Taladegib (LY2940680)	1258861-20-9	427	Piperine	94-62-2
403	Tebipenem Pivoxil	161715-24-8	428	Rutin	153-18-4
404	Bazedoxifene Acetate	198481-33-3	429	Silibinin	22888-70-6
405	Rosuvastatin Calcium	147098-20-2	430	D-Mannitol	69-65-8
406	Telotristat Etiprate (LX 1606 Hippurate)	11137608-69-5	431	L-carnitine	541-15-1
407	LY2784544	1229236-86-5	432	Sorbitol	50-70-4
408	MLN2238	1072833-77-2	433	Tolbutamide	64-77-7
409	Aliskiren Hemifumarate	173334-58-2	434	Levosimendan	141505-33-1
410	R788 (Fostamatinib) Disodium	1025687-58-4	435	Amantadine HCl	665-66-7
411	Formestane	566-48-3	436	Amfebutamone HCl	31677-93-7
412	Mubritinib (TAK 165)	366017-09-6	437	Benserazide HCl	14919-77-8
413	Irinotecan HCl Trihydrate	136572-09-3	438	Bupivacaine HCl	18010-40-7
414	Apatinib	811803-05-1	439	Bethanechol chloride	590-63-6
415	CAL-101 (Idelalisib, GS-1101)	870281-82-6	440	Chlorpromazine HCl	69-09-0
416	Eltrombopag Olamine	496775-62-3	441	Clindamycin HCl	21462-39-5
417	LY2157299	700874-72-2	442	Clonidine HCl	4205-91-8
418	Esomeprazole Sodium	161796-78-7	443	Clozapine	5786-21-0
419	Volasertib (BI 6727)	755038-65-4	444	Pramipexole	104632-26-0
420	Fesoterodine Fumarate	286930-03-8	445	Domperidone	57808-66-9
421	CX-4945 (Silmitasertib)	1009820-21-6	446	Estriol	50-27-1
422	Artemether	71963-77-4	447	Famciclovir	104227-87-4
423	Cyclosporin A	59865-13-3	448	Fluocinolone Acetonide	67-73-2
424	DL-Carnitine HCl	461-05-2	449	Gallamine Triethiodide	65-29-2
425	Nalidixic acid	389-08-2	450	Imatinib (STI571)	152459-95-5

451	Itraconazole	84625-61-6	476	Spectinomycin HCl	21736-83-4
452	Lincomycin HCl	859-18-7	477	Sulfadoxine	2447-57-6
453	Loperamide HCl	34552-83-5	478	Tenoxicam	59804-37-4
454	Manidipine	89226-50-6	479	Vardenafil HCl Trihydrate	224785-90-4
455	Manidipine 2HCl	89226-75-5	480	Xylazine HCl	23076-35-9
456	Milrinone	78415-72-2	481	Maprotiline HCl	10347-81-6
457	Mitoxantrone HCl	70476-82-3	482	Naphazoline HCl	550-99-2
458	Moroxydine HCl	3160-91-6	483	Epinephrine Bitartrate	51-42-3
459	Mycophenolic acid	24280-93-1	484	L-Adrenaline	51-43-4
460	Nateglinide	105816-04-4	485	Phenytoin sodium	630-93-3
461	Nitrendipine	39562-70-4	486	Phenytoin	57-41-0
462	Novobiocin Sodium	1476-53-5	487	Ciclopirox	29342-05-0
463	Olanzapine	132539-06-1	488	Dopamine HCl	62-31-7
464	Olopatadine HCl	140462-76-6	489	Ritodrine HCl	23239-51-2
465	Oxymetazoline hydrochloride	2315-02-8	490	Isoconazole nitrate	24168-96-5
466	Ozagrel	82571-53-7	491	Econazole nitrate	24169-02-6
467	Pancuronium dibromide	15500-66-0	492	Miconazole	22916-47-8
468	Phenoxybenzamine HCl	63-92-3	493	Secnidazole	3366-95-8
469	Propafenone HCl	34183-22-7	494	Acetanilide	103-84-4
470	Racecadotril	81110-73-8	495	Clomipramine HCl	17321-77-6
471	Ribavirin	36791-04-5	496	Phenformin HCl	834-28-6
472	Rosiglitazone maleate	155141-29-0	497	Ceftiofur HCl	103980-44-5
473	Roxithromycin	80214-83-1	498	Tiotropium Bromide hydrate	139404-48-1
474	Scopolamine HBr	114-49-8	499	Trospium chloride	10405-02-4
475	Sotalol	959-24-0	500	Tolterodine tartrate	124937-52-6

501	Sulbactam sodium	69388-84-7	526	Dimethyl Fumarate	624-49-7
502	Azelastine HCl	79307-93-0	527	Miglitol	72432-03-2
503	5-Aminolevulinic acid HCl	5451-09-2	528	Pioglitazone	111025-46-8
504	Clarithromycin	81103-11-9	529	Tolvaptan	150683-30-0
505	Rosiglitazone	122320-73-4	530	Pramiracetam	68497-62-1
506	Terbinafine HCl	78628-80-5	531	Clindamycin palmitate HCl	25507-04-4
507	Cortisone acetate	50-04-4	532	Oseltamivir Phosphate	204255-11-8
508	Amiloride hydrochloride dihydrate	17440-83-4	533	L-Thyroxine	51-48-9
509	Clomifene citrate	50-41-9	534	Gliclazide	21187-98-4
510	Cloxacillin Sodium	7081-44-9	535	Acemetacin	53164-05-9
511	Amoxicillin Sodium	34642-77-8	536	Tioxolone	4991-65-5
512	Isoprenaline HCl	51-30-9	537	Idebenone	58186-27-9
513	Medroxyprogest erone acetate	71-58-9	538	Mifepristone	84371-65-3
514	Phenylephrine HCl	61-76-7	539	Buflomedil HCl	35543-24-9
515	Prednisolone Acetate	52-21-1	540	Fluocinonide	356-12-7
516	Tetracaine HCl	136-47-0	541	Lonidamine	50264-69-2
517	Tetracycline HCl	64-75-5	542	Clorsulon	60200-06-8
518	Xylometazoline HCl	1218-35-5	543	Arecoline	300-08-3
519	Phenacetin	62-44-2	544	Noradrenaline bitartrate monohydrate	108341-18-0
520	Zidovudine	30516-87-1	545	Fostamatinib (R788)	901119-35-5
521	Quinapril HCl	82586-55-8	546	GSK2126458 (GSK458)	1086062-66-9
522	Trazodone HCl	25332-39-2	547	Ciprofibrate	52214-84-3
523	Thiamphenicol	15318-45-3	548	Dolutegravir (GSK1349572)	1051375-16-6
524	Clobetasol propionate	25122-46-7	549	Trametinib (GSK1120212)	871700-17-3
525	Brompheniramin e hydrogen maleate	980-71-2	550	Ibrutinib (PCI-32765)	936563-96-1

551	Resminostat	864814-88-0	576	S-Ruxolitinib (INCB018424)	941685-37-6
552	Nilvadipine	75530-68-6	577	Pirfenidone	53179-13-8
553	Dacomitinib (PF299804, PF299)	1110813-31-4	578	Evacetrapib (LY2484595)	1186486-62-3
554	Crenolanib (CP-868596)	670220-88-9	579	Carbazochrome sodium sulfonate (AC-17)	51460-26-5
555	TG101348 (SAR302503)	936091-26-8	580	Clevudine	163252-36-6
556	Tivantinib (ARQ 197)	905854-02-6	581	Rivaroxaban	366789-02-8
557	Varlitinib	845272-21-1	582	Paroxetine HCl	78246-49-8
558	TH-302	918633-87-1	583	Zaltoprofen	74711-43-6
559	Canagliflozin	842133-18-0	584	Pazopanib	444731-52-6
560	Dinaciclib (SCH727965)	779353-01-4	585	Amoxicillin	26787-78-0
561	Dovitinib (TKI-258) Dilactic Acid	852433-84-2	586	Aspirin	50-78-2
562	Tofacitinib (CP-690550,Tasocitinib)	477600-75-2	587	Niflumic acid	4394-00-7
563	Sotrastaurin	425637-18-9	588	Ciclopirox ethanolamine	41621-49-2
564	Sofosbuvir (PSI-7977, GS-7977)	1190307-88-0	589	Rimonabant	168273-06-1
565	Lonafarnib	193275-84-2	590	Cabazitaxel	183133-96-2
566	Galeterone	851983-85-2	591	Bufexamac	2438-72-4
567	Dabrafenib (GSK2118436)	1195765-45-7	592	Lamotrigine	84057-84-1
568	BYL719	1217486-61-7	593	Linagliptin	668270-12-0
569	Tideglusib	865854-05-3	594	Bindarit	130641-38-2
570	Clindamycin	18323-44-9	595	Vildagliptin (LAF-237)	274901-16-5
571	Carfilzomib (PR-171)	868540-17-4	596	Daunorubicin HCl	23541-50-6
572	Alogliptin	850649-61-5	597	Pravastatin sodium	81131-70-6
573	Camostat Mesilate	59721-29-8	598	Bepotastine Besilate	190786-44-8
574	Prucalopride	179474-81-8	599	Fosaprepitant dimeglumine salt	265121-04-8
575	Cobicistat (GS-9350)	1004316-88-4	600	Rofecoxib	162011-90-7

601	Cinepazide maleate	26328-04-1	626	Indacaterol Maleate	753498-25-8
602	Azilsartan	147403-03-0	627	Moguisteine	119637-67-1
603	Otilonium Bromide	26095-59-0	628	Nadifloxacin	124858-35-1
604	Bosentan Hydrate	157212-55-0	629	Pidotimod	121808-62-6
605	Rupatadine Fumarate	182349-12-8	630	Pyridoxine HCl	58-56-0
606	Azelnidipine	123524-52-7	631	Vitamin C	50-81-7
607	Alverine Citrate	5560-59-8	632	Sulfathiazole	72-14-0
608	Azilsartan Medoxomil	863031-21-4	633	Oxybutynin chloride	1508-65-2
609	Enrofloxacin	93106-60-6	634	Ornidazole	16773-42-5
610	Medetomidine HCl	86347-15-1	635	Dexamethasone Acetate	1177-87-3
611	Epinephrine HCl	55-31-2	636	Trimethoprim	738-70-5
612	Diclofenac Potassium	15307-81-0	637	Biotin (Vitamin B7)	58-85-5
613	Diclofenac Diethylamine	78213-16-8	638	Sulfamerazine	127-79-7
614	Ambroxol HCl	23828-92-4	639	Sulfamethazine	57-68-1
615	Naloxone HCl	357-08-4	640	Sodium salicylate	54-21-7
616	Chlorhexidine HCl	3697-42-5	641	Methylthiouracil	56-04-2
617	Piracetam	7491-74-9	642	Methenamine	100-97-0
618	Caspofungin Acetate	179463-17-3	643	Milnacipran HCl	101152-94-7
619	Dexmedetomidin e	113775-47-6	644	Darifenacin HBr	133099-07-7
620	Foscarnet Sodium	63585-09-1	645	Entacapone	130929-57-6
621	Tazobactam	89786-04-9, 89785-84-2	646	Estradiol valerate	979-32-8
622	Beclomethasone dipropionate	5534-09-8	647	Articaine HCl	23964-57-0
623	Atovaquone	95233-18-4	648	Gliquidone	33342-05-1
624	Etravirine (TMC125)	269055-15-4	649	Butenafine HCl	101827-46-7
625	Ulipristal	159811-51-5	650	Mepivacaine HCl	1722-62-9

651	Ethynodiol diacetate	297-76-7	676	Fexofenadine HCl	153439-40-8
652	Sertaconazole nitrate	99592-39-9	677	Moclobemide (Ro 111163)	71320-77-9
653	Tylosin tartrate	74610-55-2	678	Pergolide mesylate	66104-23-2
654	Benztropine mesylate	132-17-2	679	Cabozantinib malate (XL184)	1140909-48-3
655	Altrenogest	850-52-2	680	Sitagliptin phosphate monohydrate	654671-77-9
656	Ampicillin sodium	69-52-3	681	Lithocholic acid	434-13-9
657	Anagrelide HCl	58579-51-4	682	Ethambutol HCl	1070-11-7
658	Antipyrine	60-80-0	683	Doxycycline HCl	10592-13-9
659	Atomoxetine HCl	82248-59-7	684	Pentamidine	50357-45-4
660	Betahistine 2HCl	5579-84-0	685	Mirabegron	223673-61-8
661	Brinzolamide	138890-62-7	686	Acebutolol HCl	34381-68-5
662	Carbenicillin disodium	4800-94-6	687	Ampiroxicam	99464-64-9
663	Eletriptan HBr	177834-92-3	688	Desloratadine	100643-71-8
664	Flumequine	42835-25-6	689	Hyoscyamine	101-31-5
665	Amitriptyline HCl	549-18-8	690	Allylthiourea	109-57-9
666	Adrenalone HCl	62-13-5	691	Avanafil	330784-47-9
667	Azatadine dimaleate	3978-86-7	692	Sodium Picosulfate	10040-45-6
668	(+,-)-Octopamine HCl	770-05-8	693	Tolcapone	134308-13-7
669	Ropinirole HCl	91374-20-8	694	Probenecid	57-66-9
670	Azlocillin sodium salt	37091-65-9	695	Procaine HCl	51-05-8
671	Azacyclonol	115-46-8	696	Homatropine Methylbromide	80-49-9
672	Reboxetine mesylate	98769-84-7	697	Homatropine Bromide	51-56-9
673	Triflusal	322-79-2	698	Hydroxyzine 2HCl	2192-20-3
674	Trifluoperazine 2HCl	440-17-5	699	Aclidinium Bromide	320345-99-1
675	Meptazinol HCl	59263-76-2	700	Diphemanil Methylsulfate	62-97-5

701	Vitamin D2	50-14-6	726	Vitamin D3	67-97-0
702	Doxapram HCl	7081-53-0	727	Escitalopram Oxalate	219861-08-2
703	Dibucaine HCl	61-12-1	728	Guanabenz Acetate	23256-50-0
704	Methazolamide	554-57-4	729	Tinidazole	19387-91-8
705	Norethindrone	68-22-4	730	Guanidine HCl	50-01-1
706	Olsalazine Sodium	6054-98-4	731	Griseofulvin	126-07-8
707	Nafcillin Sodium	7177-50-6	732	Decamethonium Bromide	541-22-0
708	Tetrahydrozoline HCl	522-48-5	733	Sodium 4-Aminosalicylate	133-10-8
709	Toltrazuril	69004-03-1	734	Sodium Nitrite	7632-00-0
710	Pheniramine Maleate	132-20-7	735	Zinc Pyrithione	13463-41-7
711	Estradiol Cypionate	313-06-4	736	Propranolol HCl	318-98-9
712	Bisacodyl	30652-11-0	737	Mequinol	150-76-5
713	Carbimazole	22232-54-8	738	Mefenamic Acid	61-68-7
714	Valdecoxib	181695-72-7	739	Ticagrelor	274693-27-5
715	Valganciclovir HCl	175865-59-5	740	Triamterene	396-01-0
716	Nabumetone	42924-53-8	741	Sulfacetamide Sodium	127-56-0
717	Sertraline HCl	79559-97-0	742	Spiramycin	8025-81-8
718	Spironolactone	52-01-7	743	Lomerizine HCl	101477-54-7, 101477-55-8
719	Retapamulin	224452-66-8	744	Levobetaxolol HCl	116209-55-3, 93221-48-8
720	Methyclothiazide	135-07-9	745	Loxapine Succinate	27833-64-3
721	Ropivacaine HCl	98717-15-8	746	Flumethasone	2135-17-3
722	Sodium Nitroprusside	14402-89-2	747	Halobetasol Propionate	66852-54-8
723	Erythromycin Ethylsuccinate	1264-62-6	748	Fenspiride HCl	5053-08-7, 5053-06-5
724	Levobupivacaine HCl	27262-48-2	749	Pramoxine HCl	637-58-1
725	Ronidazole	7681-76-7	750	Difluprednate	23674-86-4

751	Droperidol	548-73-2	776	Cyclizine 2HCl	5897-18-7
752	Dydrogesterone	152-62-5	777	Dinitolmide	148-01-6
753	Halcinonide	3093-35-4	778	Clopidol	2971-90-6
754	Dexlansoprazole	138530-94-6	779	Bacitracin	1405-87-4
755	Esmolol HCl	81161-17-3, 81147-92-4	780	Azithromycin Dihydrate	117772-70-0
756	Voglibose	83480-29-9	781	Ampicillin Trihydrate	7177-48-2
757	Eprosartan Mesylate	144143-96-4	782	Amfenac Sodium Monohydrate	61618-27-7
758	Closantel Sodium	61438-64-0	783	Penfluridol	26864-56-2
759	Closantel	57808-65-8	784	Ethamsylate	2624-44-4
760	Clofazimine	2030-63-9	785	Chlorzoxazone	95-25-0
761	Estradiol Benzoate	50-50-0	786	Chlortetracycline HCl	64-72-2
762	Dicloxacillin Sodium	13412-64-1	787	Bezafibrate	41859-67-0
763	Desvenlafaxine Succinate	386750-22-7	788	Penicillin G Sodium	69-57-8
764	Desvenlafaxine	93413-62-8	789	Benzoic Acid	65-85-0
765	Triclabendazole	68786-66-3	790	Benzethonium Chloride	121-54-0
766	Histamine 2HCl	56-92-8	791	Doxofylline	69975-86-6
767	Sulconazole Nitrate	82382-23-8	792	Benzydamine HCl	132-69-4
768	Tilmicosin	108050-54-0	793	Chlorpropamide	94-20-2
769	Timolol Maleate	26921-17-5	794	Cyromazine	66215-27-8
770	Tolazoline HCl	59-97-2	795	Teriflunomide	108605-62-5
771	Sodium Phenylbutyrate	1716-12-7	796	Coumarin	91-64-5
772	Troxipide	30751-05-4	797	Choline Chloride	67-48-1
773	Clorprenaline HCl	6933-90-0	798	Cetylpyridinium Chloride	123-03-5
774	Carprofen	53716-49-7	799	Sulfaguanidine	57-67-0
775	Dropropizine	17692-31-8	800	Trometamol	77-86-1

801	Uracil	66-22-8	826	Benzocaine	94-09-7
802	Climbazole	38083-17-9	827	Montelukast Sodium	151767-02-1
803	Mezlocillin Sodium	42057-22-7	828	Dirithromycin	62013-04-1
804	Nicardipine HCl	54527-84-3	829	Valnemulin HCl	133868-46-9
805	Nifuroxazide	965-52-6	830	Liothyronine Sodium	55-06-1
806	Penciclovir	39809-25-1	831	Amoxapine	14028-44-5
807	Tiratricol	51-24-1	832	Azaperone	1649-18-9
808	Domiphen Bromide	538-71-6	833	Bosentan	147536-97-8
809	Cyclandelate	456-59-7	834	Benzbromarone	3562-84-3
810	Cinchophen	132-60-5	835	Piperacillin Sodium	59703-84-3
811	Betamipron	3440-28-6	836	Mevastatin	73573-88-3
812	Chlorquinaldol	72-80-0	837	Mexiletine HCl	5370-01-4
813	Broxyquinoline	521-74-4	838	Fidaxomicin	873857-62-6
814	Ethacridine lactate monohydrate	6402-23-9	839	Fluorometholone Acetate	3801-06-7
815	Bemegride	64-65-3	840	Oxybuprocaine HCl	5987-82-6
816	Aminothiazole	96-50-4	841	Oxaprozin	21256-18-8
817	Antazoline HCl	2508-72-7	842	Zoxazolamine	61-80-3
818	Tolperisone HCl	3644-61-9	843	Phenazopyridine HCl	136-40-3
819	Florfenicol	73231-34-2	844	Doxylamine Succinate	562-10-7
820	Furaltadone HCl	3759-92-0	845	Cetrimonium Bromide (CTAB)	57-09-0
821	Isosorbide	652-67-5	846	Deoxycorticosterone acetate	56-47-3
822	Cysteamine HCl	156-57-0	847	Serotonin HCl	153-98-0
823	Clofibric Acid	882-09-7	848	Tranylcypromine (2-PCPA) HCl	4548-34-9
824	Chromocarb	4940-39-0	849	Prucalopride Succinat	179474-85-2
825	Chlorocresol	59-50-7	850	Bromfenac Sodium	91714-93-1

851	Sulfamethoxypyridazine	80-35-3	876	Meclofenamate Sodium	6385-02-0
852	Epinastine HCl	108929-04-0	877	Salmeterol Xinafoate	94749-08-3
853	Buspirone HCl	33386-08-2	878	Mupirocin	12650-69-0
854	Luliconazole	187164-19-8	879	(R)-(+)-Atenolol	56715-13-0
855	Tamibarotene	94497-51-5	880	Anisotropine Methylbromide	80-50-2
856	Ebastine	90729-43-4	881	Dehydrocholic acid	81-23-2
857	Diacerein	13739-02-1	882	Diethylcarbamazine (citrate)	1642-54-2
858	Flufenamic acid	530-78-9	883	Diiodohydroxyquinoline	83-73-8
859	Vinorelbine Tartrate	125317-39-7	884	DL-Panthenol	16485-10-2
860	Oxiracetam	62613-82-5	885	Fluphenazine (dihydrochloride)	146-56-5
861	Rotigotine	99755-59-6	886	Halothane	151-67-7
862	Carteolol HCl	51781-21-6	887	Hexylresorcinol	136-77-6
863	Demeclocycline HCl	64-73-3	888	Piperazine	110-85-0
864	Nelfinavir Mesylate	159989-65-8	889	Sulfabenzamide	127-71-9
865	Cyclobenzaprine HCl	6202-23-9	890	Terpin (hydrate)	2451-01-6
866	Ospemifene	128607-22-7	891	Tyloxapol	25301-02-4
867	Anidulafungin (LY303366)	166663-25-8	892	Resorcinol	108-46-3
868	Micafungin Sodium	208538-73-2	893	Hydroquinone	123-31-9
869	Chloroambucil	305-03-3	894	Triacetin	102-76-1
870	MetoclopraMide HCl	7232-21-5	895	Butamben	94-25-7
871	Digoxin	20830-75-5	896	Butylparaben	94-26-8
872	Labetalol HCl	32780-64-6	897	Succinylsulfathiazole	116-43-8
873	Diphenidol HCl	3254-89-5	898	Docusate Sodium	577-11-7
874	Promethazine HCl	58-33-3	899	Amodiaquin (dihydrochloride dihydrate)	6398-98-7
875	Procainamide HCl	614-39-1	900	Nitroxoline	4008-48-4

901	Chlormadinone acetate	302-22-7	926	(+/-)-Sulfinpyrazone	57-96-5
902	Cephalothin	58-71-9	927	Chlorotrianisene	569-57-3
903	Cefazolin Sodium	27164-46-1	928	Diazoxide	364-98-7
904	Cefixime	79350-37-1	929	Prochlorperazine dimaleate salt	84-02-6
905	Lercanidipine (hydrochloride)	132866-11-6	930	Hexachlorophene	70-30-4
906	Benzyl benzoate	120-51-4	931	Isosorbide Mononitrate	16051-77-7
907	Benzyl alcohol	100-51-6	932	Sodium sulfadiazine	547-32-0
908	5-Chloro-8-hydroxy-7-iodoquinoline	130-26-7	933	Cyproheptadine hydrochloride	41354-29-4
909	Acetohydroxamic acid	546-88-3	934	Teneligliptin hydrobromide	906093-29-6
910	Gallic acid	149-91-7	935	Prasugrel Hydrochloride	389574-19-0
911	Levofloxacin hydrate	138199-71-0	936	Desogestrel	54024-22-5
912	Folic acid	59-30-3	937	Brexpiprazole	913611-97-9
913	2,2′-Dihydroxy-4-methoxybenzophenone	131-53-3	938	Lesinurad	878672-00-5
914	Diflunisal	22494-42-4	939	Tedizolid Phosphate	856867-55-5
915	Mebendazole	31431-39-7	940	Zolpidem	82626-48-0
916	Dapson	80-08-0	941	Armodafinil	112111-43-0
917	Modafinil	68693-11-8	942	Ciclesonide	126544-47-6
918	Nandrolone decanoate	360-70-3	943	Cefmenoxime hydrochloride	75738-58-8
919	Dextromethorphan (hydrobromide hydrate)	6700-34-1	944	Dantrolene sodium	24868-20-0
920	Fenoldopam (mesylate)	67227-57-0	945	Atipamezole hydrochloride	104075-48-1
921	Itopride hydrochloride	122892-31-3	946	Atipamezole	104054-27-5
922	Cefuroxime sodium	56238-63-2	947	Etoricoxib	202409-33-4
923	5,5-Dimethyloxazolidine-2,4-dione	3166-62-9	948	Sulisobenzone	4065-45-6
924	Alcaftadine	147084-10-4	949	Sulpiride	15676-16-1
925	Ethosuximide	77-67-8	950	Parecoxib	198470-84-7

951	Eslicarbazepine Acetate	236395-14-5	976	Rivastigmine	123441-03-2
952	Hydroquinidine	1435-55-8	977	Perampanel	380917-97-5
953	Nandrolone	434-22-0	978	Deoxycholic acid	83-44-3
954	Glycopyrrolate	596-51-0	979	Escin	6805-41-0
955	Tiagabine hydrochloride	145821-59-6	980	Oxybenzone	131-57-7
956	Atazanavir	198904-31-3	981	Guanfacine Hydrochloride	29110-48-3
957	Fusidate Sodium	751-94-0	982	D panthenol	81-13-0
958	Molsidomine	25717-80-0	983	ArbinoxaMine Maleate	3505-38-2
959	Rebeprazole sodium	117976-90-6	984	Saxagliptin hydrate	945667-22-1
960	Sivelestat sodium	201677-61-4	985	Tofacitinib (CP-690550) Citrate	540737-29-9
961	Lidocaine hydrochloride	73-78-9	986	Fingolimod (FTY720) HCl	162359-56-0
962	Procaine	59-46-1	987	Tacrolimus (FK506)	104987-11-3
963	Benzocaine hydrochloride	23239-88-5	988	Pimecrolimus	137071-32-0
964	Etonogestrel	54048-10-1	989	MEK162 (ARRY-162, ARRY-438162)	606143-89-9
965	Hydroxyprogeste rone caproate	630-56-8	990	Birinapant	1260251-31-7
966	Tiagabine	115103-54-3	991	Embelin	550-24-3
967	Gluconolactone	90-80-2	992	IPI-145 (INK1197)	1201438-56-3
968	Fluoxymesterone	76-43-7	993	Tepotinib (EMD 1214063)	1100598-32-0
969	Povidone iodine	25655-41-8	994	Cilengitide	188968-51-6
970	Terazosin	63074-08-8	995	LDK378	1032900-25-6
971	Protirelin	24305-27-9	996	EPZ-6438	1403254-99-8
972	Loxoprofen	68767-14-6	997	Batimastat (BB-94)	130370-60-4
973	Sildenafil Mesylate	1308285-21-3	998	Marimastat(BB-2516)	154039-60-8
974	Efavirenz	154598-52-4	999	Ilomastat (GM6001, Galardin)	142880-36-2
975	Vitamin E	59-02-9	1000	LY2835219	1231930-82-7

1001	Motolimod (VTX-2337)	926927-61-9	1026	Cerdulatinib (PRT062070, PRT2070)	1369761-01-2
1002	BAF312 (Siponimod)	1230487-00-9	1027	Lomitapide	182431-12-5
1003	Idasanutlin (RG-7388)	1229705-06-9	1028	Pilaralisib (XL147)	934526-89-3
1004	Losmapimod (GW856553X)	585543-15-3	1029	Voxtalisib (XL765, SAR245409)	934493-76-2
1005	Alvelestat (AZD9668)	848141-11-7	1030	Defactinib (VS-6063, PF-04554878)	1073154-85-4
1006	KPT-330	1393477-72-9	1031	Obeticholic Acid	459789-99-2
1007	Vidofludimus	717824-30-1	1032	LCZ696	936623-90-4
1008	Suvorexant (MK-4305)	1030377-33-3	1033	Salirasib	162520-00-5
1009	Edoxaban	1229194-11-9	1034	Dovitinib (TKI258) Lactate	915769-50-5
1010	CO-1686 (AVL-301)	1374640-70-6	1035	Elacridar (GF120918)	143664-11-3
1011	AZD9291	1421373-65-0	1036	Emricasan	254750-02-2
1012	Rilpivirine	500287-72-9	1037	Sunitinib	557795-19-4
1013	Tasisulam	519055-62-0	1038	Dasatinib Monohydrate	863127-77-9
1014	Poziotinib (HM781-36B)	1092364-38-9	1039	Erlotinib	183321-74-6
1015	Aloxistatin	88321-09-9	1040	Docetaxel Trihydrate	148408-66-6
1016	Sorafenib	284461-73-0	1041	Pexmetinib (ARRY-614)	945614-12-0
1017	Puromycin 2HCl	58-58-2	1042	Afatinib (BIBW2992) Dimaleate	850140-73-7
1018	LEE011	1211441-98-3	1043	Pexidartinib (PLX3397)	1029044-16-3
1019	Uprosertib (GSK2141795)	1047634-65-0	1044	CB1954	21919-05-1
1020	Trelagliptin	865759-25-7	1045	Eliglustat Tartrate	491833-29-5
1021	Afuresertib (GSK2110183)	1047644-62-1	1046	Ulixertinib (BVD-523, VRT752271)	869886-67-9
1022	GS-9973	1229208-44-9	1047	Tenofovir Alafenamide (GS-7340)	379270-37-8
1023	Ledipasvir (GS5885)	1256388-51-8	1048	Oltipraz	64224-21-1
1024	Filgotinib (GLPG0634)	1206161-97-8	1049	Epacadostat (INCB024360)	1204669-58-8
1025	Lomitapide Mesylate	202914-84-9	1050	Ozanimod (RPC1063)	1306760-87-1

1051	Napabucasin	83280-65-3	1054	Rocilinostat (ACY-1215)	1316214-52-4
1052	Ripasudil (K-115)	887375-67-9	1055	Otenabant (CP-945598) HCl	686347-12-6
1053	Entrectinib (RXDX-101)	1108743-60-7	1056	Empagliflozin (BI 10773)	864070-44-0
